# Numerical Evaluation and Assessment of Key Two-Phase Flow Parameters Using Four-Sensor Probes in Bubbly Flow

**DOI:** 10.3390/s25247490

**Published:** 2025-12-09

**Authors:** Guillem Monrós-Andreu, Carlos Peña-Monferrer, Raúl Martínez-Cuenca, Salvador Torró, Sergio Chiva

**Affiliations:** Department of Mechanical Engineering and Construction, Universitat Jaume I, 12071 Castelló de la Plana, Spain; carlos.pena@uji.es (C.P.-M.); rcuenca@uji.es (R.M.-C.); torro@uji.es (S.T.); schiva@uji.es (S.C.)

**Keywords:** two-phase flow, Monte Carlo simulation, four-sensor probes, local interfacial area, bubble chord length, interfacial velocity, local-flow parameter evaluation

## Abstract

Intrusive phase-detection probes remain a standard tool for local characterization of gas–liquid bubbly flows, but their accuracy is strongly affected by probe geometry and bubble–probe interaction kinematics. This work presents a Monte Carlo-based framework to evaluate four-sensor intrusive probes in bubbly flow, relaxing the classical assumptions of spherical bubbles and purely axial trajectories. Bubbles are represented as spheres or ellipsoids, a broad range of non-dimensional probe geometries are explored, and local quantities such as interfacial area concentration, bubble and flux velocities, and chord lengths are recovered from synthetic four-sensor signals. The purpose of the framework is threefold: (i) it treats four-sensor probes in a unified way for interfacial area, velocity, and chord length estimation; (ii) it includes ellipsoidal bubbles and statistically distributed incidence angles; and (iii) it yields compact correction laws and design maps expressed in terms of the spacing-to-diameter ratio $a_{p}/D$, the dimensionless probe radius $r_{p}/D$, and the missing ratio $m_{r}$ (defined as the fraction of bubbles that cross the probe footprint without being detected), which can be applied to different intrusive four-sensor probes. The numerical results show that, within a recommended geometric range $0.5\leq a_{p}/D\leq 2$ and $r_{p}/D\leq 0.25$ and for missing ratios $m_{r}\leq 0.7$, the axial velocity $V_{z}$ estimates the bubble centroid velocity and its projection with typical errors within ±10%, while a chord length correction $CL_{corr}\left(m_{r}\right)$ recovers the underlying chord length distribution with a residual bias of only a few percent. The proposed interfacial area correction, written solely in terms of $m_{r}$, remains accurate in polydisperse bubbly flows. Outside the recommended $\left(a_{p}/D,r_{p}/D\right)$ range, large probe radius or extreme tip spacing lead to velocity and chord length errors that can exceed 20–30%. Overall, the framework provides quantitative guidelines for designing and using four-sensor intrusive probes in bubbly flows and for interpreting their measurements through geometry-aware correction factors.

## 1. Introduction

Intrusive sensor probe techniques are commonly used in two-phase flow experiments for the measurement of time-averaged principal local flow parameters concerning the disperse phase, such as void fraction, bubble velocity and frequency, bubble size, and interfacial area concentration. These parameters play an important role in modeling the dynamic behavior of the bubbles, for example, the drag force, lift force, or virtual mass force. Additionally, the current development of Eulerian or Lagrangian 3D thermal–hydraulic codes requires local measurement data from real facilities to validate current models and simulation results.

The accurate measurement of local parameters such as bubble velocity and interfacial area concentration (defined as the total interfacial area per unit mixture volume) is crucial for modeling mass, momentum and energy transfer in gas–liquid two-phase flows. Small deviations in bubble velocity measurements translate into large uncertainties in the relative velocity or slip ratio, which directly affect the evaluation of interfacial momentum transfer. Likewise, the interfacial transfer mechanisms are controlled by the total available interfacial area, as mass, momentum, and energy are exchanged between phases through the gas–liquid interface. This role is made explicit in the local instantaneous conservation equations for two-phase flow given by [1], where the interfacial area concentration $a_{i}$ governs the interfacial source terms.

Within this context, the two-fluid model of [2] remains one of the most conceptually advanced frameworks for two-phase flow analysis, as it can describe transient and developing conditions while treating phase interactions at the interface. In the two-fluid formulation, appropriate constitutive relations for the interfacial transfer terms are needed to close the phase balance equations, and their accuracy strongly influences the model predictions. Since these interfacial transfer terms are typically proportional to $a_{i}$, the reliability of interfacial area measurements is a key prerequisite both for robust two-fluid simulations and for a deeper understanding of the thermo-hydraulic behaviour of multiphase flows.

Unfortunately, experimental data from two-phase flows are difficult to obtain and validate due to their inherent stochastic behavior. In most cases, direct non-intrusive optical imaging techniques cannot be applied because high void fractions impede the optical path. In this sense, intrusive instrumentation is the most appropriate and accurate way to obtain local information from two-phase flow experiments. Intrusive sensor probes provide access to local properties of the flow while minimizing intrusive effects if a small size can be achieved in their design and construction.

Single- [3,4], double- [5,6,7,8], and four-sensor probes [9,10,11] have been developed in past decades and have been successfully tested to measure cited local flow parameters. Although void fraction and bubble frequency are “direct” measurements obtained from sensor probes because they only require the information provided by one sensor tip [12], velocity and interfacial area measurements require flow assumptions and corrective factors to be properly evaluated [13,14]. Moreover, due to the complexity of the motion and geometry of the interface of the bubbles, the measurement of interfacial area is difficult, evidenced by the lack of existing data in the literature. In this sense, the four-sensor probe method is a useful measurement technique for flow parameters, especially designed for local interfacial area measurements.

The objective of this work is the generalization of the four-sensor measurement technique by means of numerical simulations, which would provide the theoretical probe limits (size and spatial distribution of the sensor tips) and necessary correction factors, tested in a wide variety of two-phase flow scenarios. To accomplish this task, we have developed a numerical framework to test virtual four-sensor probes and obtain the main local flow parameters with the same procedures as in real experiments. We will also present a conceptual discussion about the possible methods and their limitations to recover and evaluate the two-phase flow local parameters.

The new numerical framework presented in this work is conceptually different from previous methods [13,14,15,16], aiming to provide useful theoretical limits and corrections for practical two-phase flow measurements using four-sensor probes, such as local interfacial area concentration, chord length, bubble velocity, and flow rate validation. Although it is a different framework concept, many of the presented results are in accordance with previous studies, especially with previous work from Le Corre and Ishii [14] in terms of interfacial area recovery. Beyond these contributions, the present work offers a generalized assessment framework that complements and extends previous theoretical and numerical studies. First, while Ishii’s [17] interfacial area transport theory and subsequent developments provide the macroscopic balance laws and source terms, here we focus on how local four-sensor measurements can be rigorously connected to those quantities at the scale of individual bubble–probe interactions. Second, compared to the revolutionary four-sensor methodology by Kataoka et al. [18], which provides the mathematical basis to reconstruct the interface-normal velocity and therefore the local interfacial area, the present framework explicitly explores a broader space of probe geometries (square and tetrahedral), sensor spacing-to-diameter ratios, and near-wall configurations, quantifying their impact on measurement bias. Third, while Le Corre and Ishii [14] and later authors used Monte Carlo simulations to examine probe–bubble interactions and derive interfacial area corrections under the assumption of spherical bubbles and nearly axial incidence, our framework relaxes these assumptions by incorporating ellipsoidal bubble shapes and statistically distributed incidence angles, and by systematically comparing centroidal axial velocity, interface-normal velocity and chord length estimates. Finally, we provide compact correction factors and practical design guidelines expressed in terms of the missing ratio and non-dimensional probe geometry, which are directly applicable to existing four-sensor probes in bubbly flow conditions. In this sense, the framework is “conceptually new” in that it treats the probe as a generalized measurement operator acting on a synthetic bubbly field, enabling a unified evaluation of interfacial area, bubble velocity, and chord length measurements across a wide range of geometries and flow anisotropies.

The present work is divided into eight sections. Theoretical background for the local flow measurements and four-sensor probe principle of operation is presented in Section 2. In Section 3, the numerical framework used for simulations is described. Section 4 presents a comprehensive discussion and significance of all two-phase flow parameter definitions available from simulations. Section 5 consists of a brief review of previous works and the specific parameters and organization of numerical simulations performed and used in this work. Results are presented in Section 6 and Section 7. Section 6 focuses on the effect of probe geometry (sensor tip spatial arrangement) over measurable two-phase flow parameters towards establishing a general probe design criteria. Section 7 provides the main results of the theoretical performance of the four-sensor probe in bubbly flow, including specific scenarios such as near-wall measurements, and finally, the conclusions are given in Section 8.

## 2. Four-Sensor Probes

**Figure 1:**
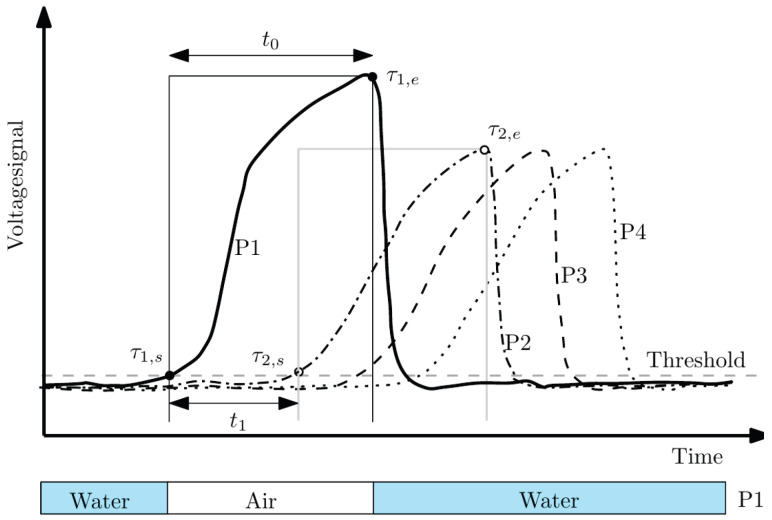
Typical bubble voltage signature signals from air–water two-phase flow.

The measurement principle of double-sensor and four-sensor probes is based on phase discrimination at the probe tip end (local measurement). Optical probes depend on the critical angle for reflection/refraction changes in the different phases. For conductivity probes, the discrimination is based on the large change in medium conductivity. Each type of probe is limited by its principle to operate with mixtures, usually used only with two phases, with marked specific property changes between them (conductivity and refraction index). The signals obtained are very similar, thus, the signal processing methods are almost equivalent. Local, time-averaged two-phase flow parameters using multi-sensor probes are based on the phase discrimination voltage signal (using the differences in electrical or optical properties between the phases), obtained simultaneously from each sensor of the probe. Figure 1 shows typical signals corresponding to a bubble–sensor interaction event from a four-sensor probe, in this case, obtained from a four-sensor conductivity probe in an air–water mixture [19].

A low signal level indicates water, whereas a high level indicates air. Ideally, the sensor response is 1 inside the gas and 0 inside the liquid, but real signals differ from an ideal two-state response, caused by differences in the wetting/de-wetting process while bubble probe tips are passing through the interface boundary [20,21]. This recovery or transformation into a binary signal is performed by means of a threshold level [3,12]. From the pulse duration and time delays between signals, it is possible to obtain the main local flow parameters regarding the disperse phase. For each bubble–probe passage, as depicted in Figure 1, a set of times are obtained for the front bubble interface (subscript *s*) and for the rear interface (subscript *e*), defined as follows:

Front tip (1): [$\tau_{1,s}$, $\tau_{1,e}$], with $\tau_{1,s}<\tau_{1,e}$
Rear tip (2): [$\tau_{2,s}$, $\tau_{2,e}$], with $\tau_{2,s}<\tau_{1,s}$ and $\tau_{2,e}>\tau_{2,s}$
Rear tip (3): [$\tau_{3,s}$, $\tau_{3,e}$], with $\tau_{3,s}<\tau_{1,s}$ and $\tau_{3,e}>\tau_{3,s}$
Rear tip (4): [$\tau_{4,s}$, $\tau_{4,e}$], with $\tau_{4,s}<\tau_{1,s}$ and $\tau_{4,e}>\tau_{4,s}$

At time $\tau_{1,s}$, the front tip reaches the bubble interface. Taking $\tau_{1,s}$ as a reference time for the interaction event and considering only the first incoming interface, we can completely define this interaction based on a set of four time differences:

(1) $$\Phi =(t_{0},t_{12},t_{13},t_{14}),$$

where $t_{0}=\tau_{1,e}-\tau_{1,s}$ and $t_{1i}=\tau_{i,s}-\tau_{1,s}$ for i=2,…,4.

**Figure 2:**
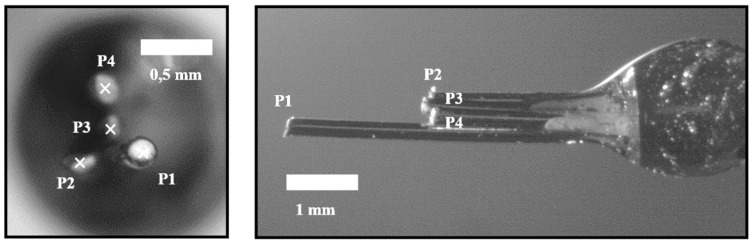
Detailed view of a four-sensor conductivity probe’s tips: radial view (**left**) and axial view (**right**).

A typical four-sensor probe is depicted in Figure 2. Three rear sensors (P2, P3, and P4) are mounted around a central sensor (P1).

Usually, the probe is oriented in the main flow direction, and therefore P1 is considered the front or reference sensor, as it will perform the earliest detection of the bubble interface. For two-phase local flow parameter calculation, the spatial location of the rear sensors’ tips is always related to the spatial location of the front sensor tip.

Provided the four points in space correspond to the location of the four sensor tips (P1, P2, P3, and P4) and signal time delays (Φ), it is possible to compute the main two-phase flow parameters, according to definitions depicted in the following section.

### 2.1. Time-Averaged Local Parameters Measured with Needle Sensors

**Figure 3:**
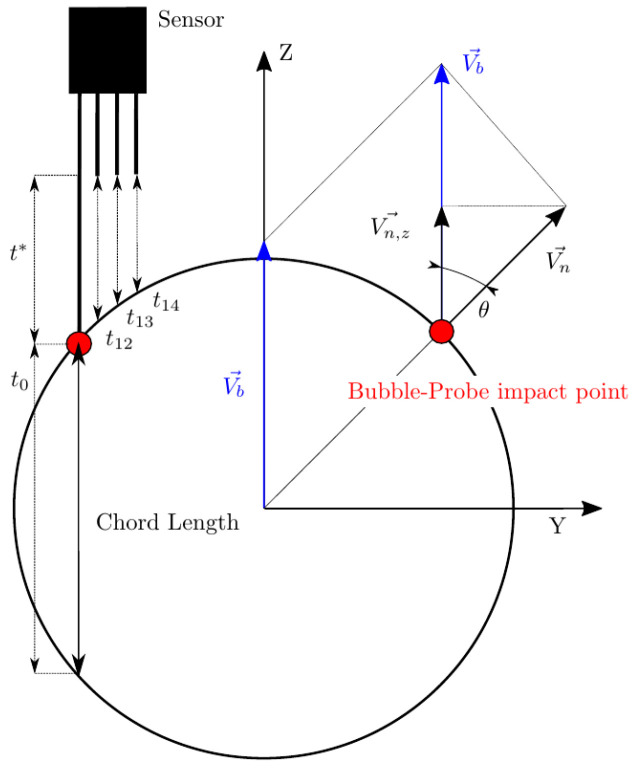
Schematic of available measurements from four-sensor conductivity probe.

As a first step before introducing the complete simulation framework, presented in the next section, we will define the principal measurable variables by means of the signal obtained from one upstream and one or three downstream sensors. Considering the information included in Figure 3, the following local time-averaged two-phase flow parameters can be defined:

#### 2.1.1. Local Time-Averaged Void Fraction α

Defined as the ratio of gas-phase total residence time (τ) to the total measurement time interval (*T*).

(2) $$\alpha \left(x,y,z\right)=\frac{\tau }{T}=\frac{1}{T}\sum_{i=1}^{N_{b}}t_{0,i}$$

It is computed as the time-averaged summation of the individual residence time ($t_{0}$) from all bubbles pierced by the front sensor ($N_{b}$). The void fraction associated with each *i*-bubble ($t_{0,i}$) is a necessary measurement to obtain the rest of the local flow parameters defined below. It is also an important parameter since it describes the proportion of phases locally.

#### 2.1.2. Local Time-Averaged Interfacial Area Concentration $a_{i}$

From the general definition of volumetric interfacial area, defined as the ratio of available surface per unit area, Ishii [17] introduced the concept of local time-averaged interfacial area applicable to local sensors. It was defined as the sum of the reciprocals of the speed displacement of each interface (or interfacial velocity) passing through a point in the space divided by the time considered for the summation:

(3) $$a_{i}\left(x,y,z,t\right)=\frac{1}{T}\sum_{j=1}^{2N_{b}}\frac{1}{|\vec{V_{b,j}}\cdot \vec{n_{j}}|}$$

where the summation is performed over the $2N_{b}$ (front and rear) bubble interfaces passing through an arbitrary location at point P(x,y,z) during a *T* time interval. As discussed by Morel and Delhaye in [22], the correct interpretation for interfacial velocity could lead to erroneous interpretations, and should be understood only as a velocity to compute the local interfacial area, not as a reliable velocity to describe the bubble movement, since it depends entirely on the surface point where it is defined. More extensive discussions about bubble velocity measurements will be performed in the following sections.

At first glance, it is difficult to relate the volumetric and local definition of interfacial area concentration. Letting *V* be a fixed control volume and $A_{i}$ the sum of all area of the interfaces enclosed in *V*, the instantaneous, global interfacial area concentration Γ(t) is defined as

(4) $$\Gamma \left(t\right)=\frac{A_{i}}{V}$$

Meanwhile, the local instantaneous interfacial area concentration, as it appears in the local formulation of mass, momentum, and energy conservation equations of two-phase flows [1], is mathematically defined by [1,17,23]:

(5) $$a_{i}\left(x,y,z,t\right)=\left|\nabla f\right|\delta \left(f\left(x,y,z,t\right)\right)$$

where the function f(x,y,z,t)=0 represents the interface detection and δ is the delta function. So, in practice, Equation (5) indicates that to compute each bubble interfacial area contribution in a given period, it is necessary to define the properties of all points located in the surface. Those properties are the orientation of the interface (normal to surface unity vector, linked to surface gradient at a given point) and its instantaneous transport velocity or interfacial velocity (differential displacement along gradient vector).

Delhaye [24] demonstrated the relationship between the time-averaged instantaneous global interfacial, Γ(t), and the local volume-averaged interfacial area concentration, $a_{i}\left(P,t\right)$, at the given space point P(x,y,z):

(6) $$\frac{1}{V}\int_{V}a_{i}dV\equiv \frac{1}{T}\int_{T}\Gamma dt$$

Considering a given point P(x,y,z) in volume *V*, each time an interface passes through P(x,y,z) it has a speed of displacement $\vec{V_{b,j}}\cdot \vec{n_{j}}$ where $\vec{V_{b,j}}$ stands for the bubble velocity and $n_{j}$ the unit vector normal to the interface at that local measurement point. It was shown by Delhaye [25] that the following identity holds:

(7) $$\frac{1}{V}\int_{V}\frac{1}{T}\sum_{j}\frac{1}{|\vec{V_{b,j}}\cdot \vec{n_{j}}|}dV\equiv \frac{1}{T}\int_{T}\Gamma dt$$

where *j* denotes the *j*-th interface passing through P(x,y,z) during the time interval *T*. The integrand of the left-hand equation is the same local time-averaged definition for interfacial area concentration introduced by Ishii in Equation (3). This definition is very useful for needle-sensor probe methodologies, as it can recover the local interfacial area value only by means of interfacial velocity. Moreover, this relationship between global and local interfacial area will be very useful for testing the convergence of numerical simulations presented in the following sections.

The practical application of Equation (3) relies on the type of the sensor probe to be used. Depending on the number of probe tips, we can divide intrusive sensors into two types: double-sensor and four-sensor probes. The main difference between these sensors is the method used to compute the interfacial velocity considered in Equation (7). For double-sensor probes, Equation (3) can be rewritten as:

(8) $$a_{i}\left(x,y,z,t\right)=\frac{1}{T}\sum_{j=1}^{2N_{b}}\frac{1}{|\vec{V_{b,j}}\cos\theta_{j}|}$$

where $\theta_{j}$ refers to the angle between the measured velocity and the normal to surface vector at point P(x,y,z) for *j*-th interface. The problem is that a double-sensor probe only provides unique velocity measurement, aligned with the sensor probe axis, and thus $\theta_{j}$ can not be directly measured. This means that several assumptions should be made to estimate the normal to surface velocity component. These assumptions are spherical bubbles and negligible radial bubble movement, or a correction factor to evaluate the $\cos\theta_{j}$ term, as in [26].

Before introducing the formal expressions for a four-sensor probe, it is useful to recall the underlying physical picture. As a bubble crosses the vicinity of the probe, each tip records a binary phase-detection signal that switches from liquid to gas when the interface first intersects its sensing volume, and back to liquid when the interface leaves. For a four-sensor arrangement, these four switching times define a tetrahedron in space–time: the spatial positions of the tips and the corresponding arrival times of the interface. Under the assumption that the local interface can be approximated by a plane moving at constant velocity over the scale of the probe, the intersection between this plane and the four sensing points fully determines both the orientation of the interface (through its normal vector) and its propagation velocity. The classical four-sensor relations by Kataoka et al. [18] exploit precisely this geometrical construction: the interface-normal velocity, $V_{n}$, is reconstructed from the determinant of the time differences, while the local interfacial area density follows from counting the number of interface crossings per unit time and unit area. It takes advantage of the use of three rear sensors to calculate the bubble velocity projection over the normal to surface vector, and so can provide an accurate estimation for $\theta_{j}$. Local interfacial area can be computed only by means of geometrical parameters and time delays between the front and rear tips of the probe:

(9) $$a_{i}=\frac{1}{T}\sum_{j=1}^{2N_{b}}\frac{\sqrt{{D_{1j}}^{2}+{D_{2j}}^{2}+{D_{3j}}^{2}}}{{D_{0}}^{2}}$$

where *i* represents a single bubble front interface and the relative position of the *j*-rear sensors (j=2,3,4) with respect to the front sensor is represented as $x_{1j}$, $y_{1j}$, and $z_{1j}$:

(10) $$D_{0}=\left|\begin{array}{ccc} x_{12} & y_{12} & z_{12}\\ x_{13} & y_{13} & z_{13}\\ x_{14} & y_{14} & z_{14} \end{array}\right|\;\;\;\;D_{1j}=\left|\begin{array}{ccc} t_{12} & y_{12} & z_{12}\\ t_{13} & y_{13} & z_{13}\\ t_{14} & y_{14} & z_{14} \end{array}\right|\;D_{2j}=\left|\begin{array}{ccc} x_{12} & t_{12} & z_{12}\\ x_{13} & t_{13} & z_{13}\\ x_{14} & t_{14} & z_{14} \end{array}\right|\;\;\;\;D_{3j}=\left|\begin{array}{ccc} x_{12} & y_{12} & t_{12}\\ x_{13} & y_{13} & t_{13}\\ x_{14} & y_{14} & t_{14} \end{array}\right|$$

So, according to Equation (9), the local interfacial area concentration only depends on probe geometry and the time delays from the probe tip signal. The only assumption in Equation (9) is that the sensor probe should be small compared to bubble size in order to consider the surface as a tangent plane [18,27]. In fact, this means that the probe measurements could be influenced by local bubble curvature. Therefore, the size and the geometry of the sensor probe should be carefully determined.

#### 2.1.3. Measurable Velocities by Means of a Four-Sensor Probe

Considering an ideal double-sensor probe (both sensors perfectly axially aligned), individual *i*-bubble velocity measurement could be estimated from the signal’s time delay (flight time of the detected interface between front and rear sensor) and the sensors’ axial spacing:

(11) $$V_{z,i}=\frac{\Delta S}{\Delta t}=\frac{z_{12}}{t_{12}}$$

However, there is no straightforward way to distinguish between possible measurable bubble velocities: velocity can refer to the bubble centroid velocity, $V_{b}$ (Figure 3), or to the interface, $V_{n}$ (interfacial velocity).

Four-sensor probes provide two extra measurements from added rear sensors which can help to distinguish between measurable velocities. One common method to evaluate $V_{b}$ (equivalent to Equation (11)) for four-sensor probes is using the averaged information provided by the rear sensors, as proposed in [28]:

(12) $$V_{z,i}=\frac{1}{3}\sum_{j=2}^{4}\frac{z_{1j}}{t_{1j}}$$

In [28], $V_{z}$ is considered as the interfacial velocity parallel to the sensor probe axis. It could lead to erroneous interpretations, as $V_{z}$ is not related to interfacial velocity but to bubble centroid velocity projection to the sensor probe axis. Strictly, the interfacial velocity parallel to sensor probe axis is $V_{n,z}$, related to $V_{b}$ by a $\cos^{2}\theta$, as depicted in Figure 3.

Four-sensor probes can provide, under some assumptions, a direct measurement for $\vec{V_{n}}$ (and also for its components, ($\vec{V_{n,x}}$, $\vec{V_{n,y}}$, and $\vec{V_{n,z}}$)) derived from Equation (9), as proposed by [27]:

(13) $$\vec{V_{n,j}}=\frac{{D_{0}}^{2}}{\sqrt{{D_{1j}}^{2}+{D_{2j}}^{2}+{D_{3j}}^{2}}}$$

It is important to keep in mind that sensor probes are not ideal, so radial distances between sensors should be considered. As depicted in Figure 3, the ideal time delay considering ideal sensor probes ($t^{*}$) differs from real measurable time delays ($t_{12}$, $t_{13}$, and $t_{14}$), which are affected by the local curvature. This fact could also lead to erroneous evaluation of measurable velocities and also of interfacial area (as it is directly related to interfacial velocity).

It should be emphasized that the above relations describe an idealized probe response in which the phase-detection signal switches instantaneously at a well-defined interface crossing. Real probes are affected by additional error sources that are not explicitly included in the present Monte Carlo framework, such as partial wetting of the sensor surface, hysteresis in the switching due to finite sensor response time, electronic noise, and the choice of a specific voltage or conductivity threshold for phase discrimination. These effects mainly alter the effective detection time at each tip and can introduce additional jitter or bias in the reconstructed arrival times. In practice, such instrumental and signal-processing uncertainties are often treated separately, for instance by optimizing the threshold or filtering strategy. In this work, we intentionally neglect them to isolate the purely geometrical and kinematic contributions to the measurement bias; the resulting correction factors and design guidelines can then be combined with standard signal-conditioning procedures in real applications.

#### 2.1.4. Chord Length

Individual chord length ($CL_{j}$) is obtained as the product of each bubble flight time from the front sensor tip ($t_{0,j}$) and bubble centroid velocity ($V_{b}$). Ideally, chord length defined as in Figure 3 can only provide a rigorous estimation of the real mean bubble diameter ($D_{b}$) under the assumption of monodisperse spherical bubbly flow with bubble velocity always parallel to sensor probe [29]. Estimation of $D_{b}$ from chord length can be biased due to lateral motion of the bubbles, non-uniform bubble size population, or if bubble velocity is not correctly measured. Extensive efforts can be found in the literature to relate the chord length distribution to bubble-size distribution in complex scenarios [30,31,32,33,34]. The main problem is that the chord length is measured ideally directly as a distance without considering real sensor error measurements or limitations. Experimentally, the chord length should be evaluated as the product of *i*-bubble velocity $V_{b}$ and the associated time lapse between the detection of the front and rear interface, $t_{0,i}$. Therefore, it is very important to consider carefully how the bubble velocity is defined and measured. Regarding this issue, one of the goals of the present work is to evaluate the four-sensor probe’s capability to generate the chord length probability distributions from a given simulated bubble population and evaluate the possible deviations.

## 3. Simulation Framework

**Figure 4:**
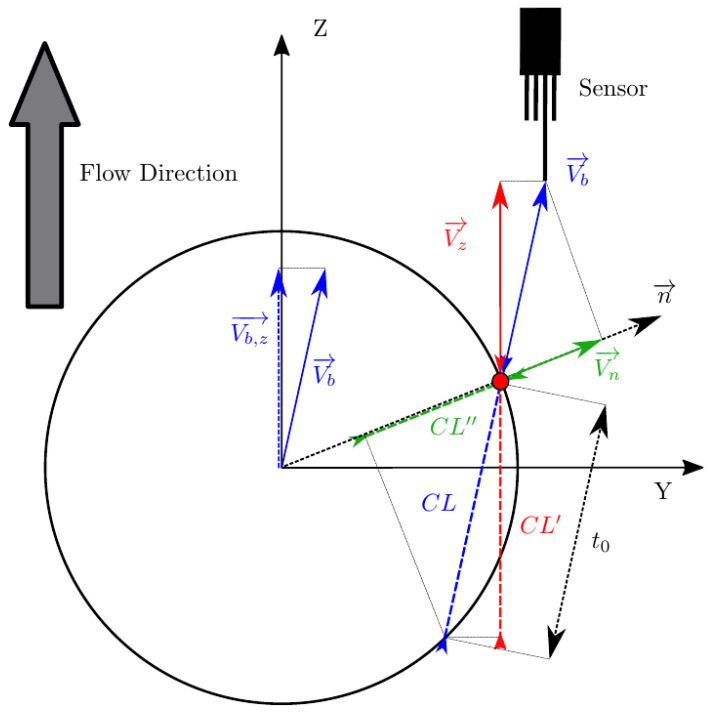
Measurable bubble velocities and chord lengths in a bubble–probe interaction. General case.

In real scenarios, bubble velocity depends on several flow conditions (i.e., liquid-phase velocity and turbulence intensity), thus, bubble movement is rarely aligned with the sensor probe as depicted in Figure 3, which illustrates the simplest and ideal scenario, where a bubble with no radial velocity fluctuation moves parallel to a sensor probe. But, even in this case, it is clear that bubble velocity and interfacial velocity are not directly related. A more general and realistic scenario is depicted in Figure 4, where bubble velocity is not aligned with the main flow direction or in the sensor axis direction.

The real or exact chord length (CL) is computed from $\vec{V_{b}}$ and the time $t_{0}$ computed with the front sensor. This fact highlights two important limitations of needle probes:

- To date, from the available methods to recover bubble velocity by using four-sensor probes (Equations (12) and (13)), we cannot account for the bubble centroid velocity, $\vec{V_{b}}$. Four-sensor probes are limited to two velocity modulus projections measurements, along the sensor probe axis, $V_{z}$, or along the normal to surface direction, $V_{n}$, and its components.
- As depicted in Figure 4, real or exact bubble chord length is measured in the bubble centroid velocity direction as $CL=|\vec{V_{b}}|t_{0}$. The sensor probe CL measurements are limited to $CL^{\prime }=\left|\vec{V_{z}}\right|t_{0}$ and $CL^{\prime \prime }=\left|\vec{V_{n}}\right|t_{0}$. In the ideal case, considering all measurement error sources to be negligible, we will always obtain $CL^{\prime \prime }\leq CL^{\prime }\leq CL$.

Therefore, unless we consider the highly simplified scenario as depicted in Figure 3, where $\vec{V_{b}}=\vec{V_{b,z}}=\vec{V_{z}}$, exact chord length cannot be correctly estimated by means of $\vec{V_{z}}$ measurement. Furthermore, the exact CL recovery of $\vec{V_{n}}$, $CL^{\prime \prime }$, is not possible as it depends on the interfacial orientation at the contact point, defined by $\vec{n}$.

**Figure 5:**
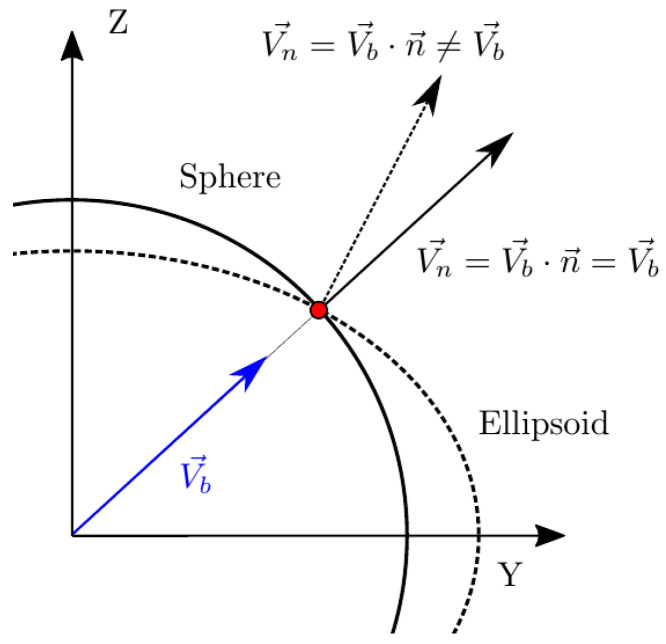
Bubble geometry definition effect in the local interfacial velocity.

In [6,13,14], the authors simulated variability in bubble–probe interactions by means of changes in the direction and modulus of a bubble velocity vector. However, the orientation of the sensor with respect to the bubble coordinate reference system was kept constant. In practice, if bubble sphericity is assumed, it is equivalent to consider that the bubble velocity equals its interfacial velocity in their numerical simulations. These assumptions are fairly correct, since their objective was to recover and study the interfacial area obtained by means of double- and four-sensor probes, which is in fact only dependent on interfacial velocity (Equation (3)). However, these assumptions do not provide the necessary framework to evaluate four-sensor probe performance in terms of bubble velocity or chord length measurement without spherical assumption. As illustrated in Figure 5, for spherical geometries, local interfacial velocity can be defined equivalently as a bubble centroid velocity. However, for ellipsoidal geometries, local interfacial velocity depends on the local curvature, so it cannot be related to bubble centroid velocity.

**Figure 6:**
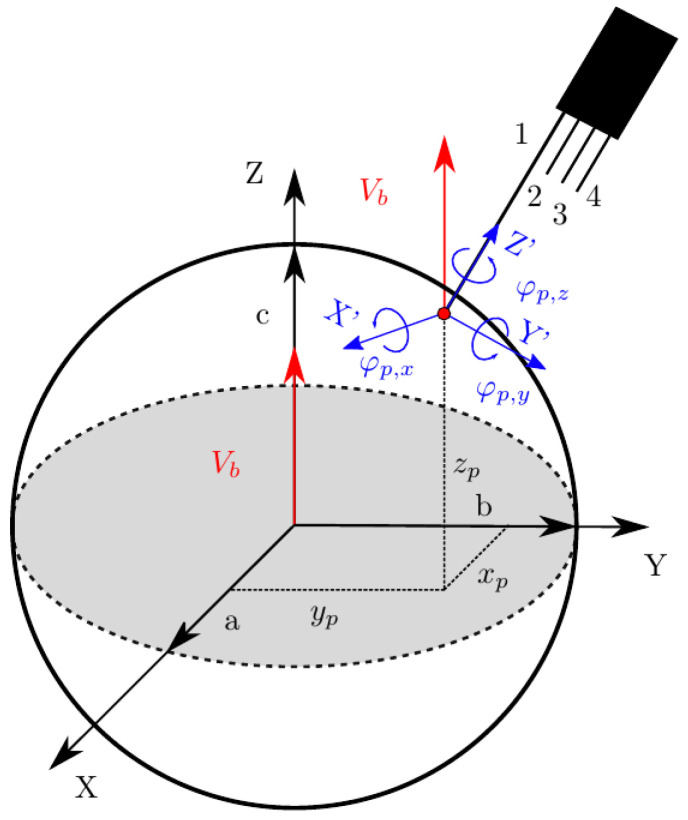
Single bubble-to-probe interaction.

The present work aims to provide a general framework where no assumption is made on bubble shape or bubble centroid velocity to interfacial velocity, thus providing the necessary tool to evaluate the accuracy of velocity, interfacial area, and chord length measurements independently. Figure 6 illustrates the framework to be used, where the main reference Cartesian coordinate system (XYZ) is located at the origin of a bubble, and an auxiliary Cartesian coordinate system (X′Y′Z′) for the four-sensor probe is located at the front sensor tip.

Bubbles are considered “frozen” (as long as they cannot change orientation) and their displacement restricted to the Z-axis direction. However, the probe can freely rotate with respect to the XYZ coordinate system. Orientation changes and relative position for probe–bubble interactions only depend on changes (relative translation or rotation) in X′Y′Z′ with respect to XYZ. We consider an ellipsoidal bubble with the arbitrary axes *a*, *b*, and *c*. Thus, the bubble surface is defined as

(14) $$\frac{x^{2}}{a^{2}}+\frac{y^{2}}{b^{2}}+\frac{z^{2}}{c^{2}}=1$$

We also consider that a single interaction event *n* between a bubble and the four-sensor probe can be geometrically described by a parametrized model ψ, providing the four representative time delays, Φ:

(15) $$\psi_{n}\left(a,b,c,V_{b},x_{p},y_{p},r_{p},a_{p},\varphi_{p,x},\varphi_{p,y},\varphi_{p,z}\right)\to \Phi_{n}$$

Variables included in the parametrized model are depicted in Figure 6, where

- $V_{b}$ is the modulus of bubble centroid velocity, always defined as aligned in the Z-axis direction.
- $a_{p}$ and $r_{p}$ are, respectively, the axial and radial distances of the rear sensor tips with respect to the front sensor axis. We will relate these parameters to bubble equivalent diameter, *D*, defined as $D=2\sqrt[{3}]{abc}$.
- $\varphi_{p}=\left[\varphi_{p,x}\;\varphi_{p,y}\;\varphi_{p,z}\right]$ represents the rotation angle vector in the probe coordinate system. $\varphi_{p,x}$ and $\varphi_{p,y}$ determine the arbitrary orientation of the probe sensor, while $\varphi_{p,z}$ allows an arbitrary rotation of the rear sensors around the front sensor axis.
- $x_{p}$ and $y_{p}$ define the position of the front sensor tip in the XY plane.

### Algorithm and Monte Carlo Simulation

Monte Carlo simulation is a technique used to study how a model responds to randomly generated inputs. In the presented framework, we will simulate an aggregate of single bubble–probe interactions with arbitrary bubble geometry and arbitrary positioning of the sensor probe with respect to the fixed bubble reference coordinate system. The principal benefit of this method is to reproduce the information from a real experimental condition (Φ) by means of virtual four-sensor probes given perfectly defined bubble population characteristics (size and velocity distributions). Therefore, this approach allows us to assess multiple error sources and probe geometrical theoretical operating limits, information which could have a direct application in real experiments.

**Figure 7:**
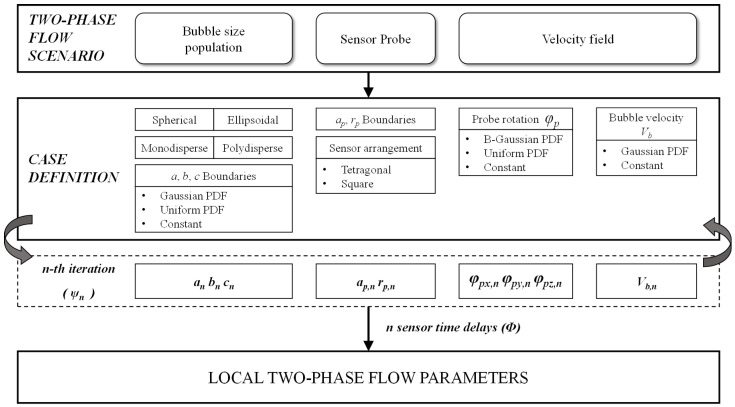
Numerical simulation methodology.

In order to closely reproduce multiple two-phase flow conditions, we controlled the variability of the variables included in ψ, as depicted in Figure 7.

As a first step, bubble population characteristics are selected. We can choose between monodisperse or polydisperse bubble populations, where bubbles can be randomly generated as spheroids and/or ellipsoids, considering minimum and maximum size ranges of variability independently for the three bubble principal axes. For polydisperse bubble generation, bubble sizes are generated from Gaussian or uniform distribution.

Control parameters, $r_{p}/D$ and $a_{p}/D$, are defined from the equivalent diameter of bubble distribution generated and randomly generated values for $r_{p}$ and $a_{p}$, from a uniform probability distribution. These control parameters will provide insight about the accuracy of two-phase flow parameter measurements given four-sensor probe geometry. In typical probe designs, the diameters (optical or conductivity) of probe sensor tips are usually about 100–300 μm and typical bubble diameters are about 2–6 mm. In our simulations, we will consider *a*, *b*, *c*∈ [1, 3] mm for bubble size generation (D∈ [2, 6] mm). In addition, we include in our model the spatial arrangement of rear sensors (teragonal or square geometries, defined in Section 6).

We introduce the bubble velocity field variability by two methods. First, although the bubbles are set up in a “frozen” coordinate system XYZ, the modulus of individual bubble velocity ($V_{b}$) can be set as constant (unitary velocity) or obtained from a Gaussian probability distribution with mean equal to one and random standard deviation obtained from a uniform probability distribution within the interval [0, 1/3]. Second, complementary to the first method, is by the spatial rotation of the four-sensor probe, defined by $\varphi_{p}$. The main advantage of this approach is that we can easily control the sources of bubble velocity variability separately.

**Figure 8:**
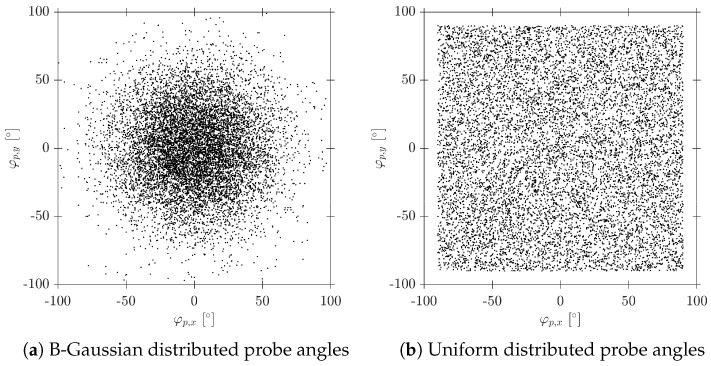
Probe inclination angle definitions with $\varphi_{p,max}=90^{{}^{\circ}}$.

Probe inclination angles $\varphi_{p,x}$ and $\varphi_{p,y}$ in the sensor probe coordinate system are obtained from a Bivariant Gaussian distribution with zero mean and standard deviation equal to $\varphi_{p,max}/3$, with $\varphi_{p,max}$ being the maximum bubble centroid attacking angle, set as constant or obtained from a uniform probability distribution with limits [0°, $\varphi_{p,max}$], as depicted in Figure 8.

The probe inclination angles $\varphi_{p,x}$ and $\varphi_{p,y}$ qualitatively account for bubble radial fluctuations: $\varphi_{p,max}=0^{{}^{\circ}}$ simulates a scenario where all bubble velocities are parallel to the probe axis (perfect vertical flow), while $\varphi_{p,max}=90^{{}^{\circ}}$ includes bubble velocities perpendicular to the probe axis, and the $\varphi_{p,z}$ rotation angle accounts for the rotation of the probe around its Z’-axis. $\varphi_{p,z}$ rotation is used as an extra source of variability, as it allows for a different tip orientation at each bubble–probe interaction event.

**Figure 9:**
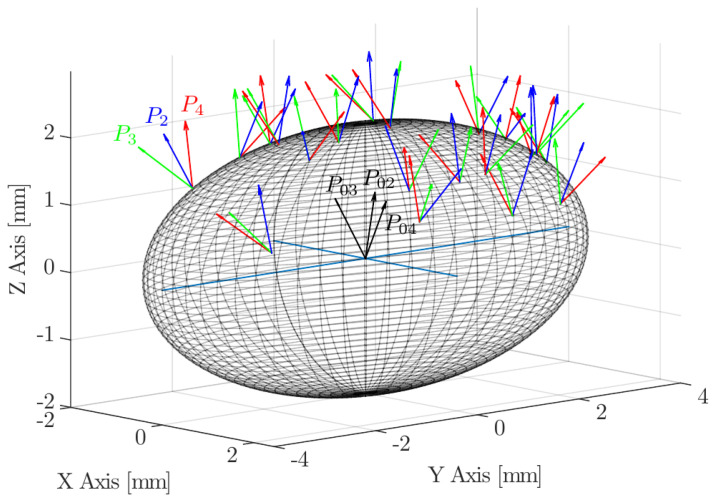
Example of sensor probe random re-orientation for 20 sample points.

Therefore, once excepting the variables in $\psi_{n}$, the *n*-th bubble–probe interaction is defined according to the following steps:

1. Sensor alignment is initialized coincident to the Z-axis and with the front sensor tip located at the XYZ origin. Each *k*-th (k=2,3,4) rear sensor tip’s initial position ($P_{0k}=\left[x_{0k},y_{0k},z_{0k}\right]$) is referenced to the front sensor tip and defined by $a_{p}$ and $r_{p}$ distances as in Table 1. The front sensor tip will be positioned at a surface point of the bubble, defined by $x_{p}$, $y_{p}$, and $z_{p}$, thus representing the start time of a bubble–probe interaction. The location $z_{p}$ is directly obtained from Equation (14) and locations $x_{p}$ and $y_{p}$ are obtained from a uniform distribution with the respective limits $x_{p}\in \left[-a,a\right]$, $y_{p}\in \left[-b,b\right]$ and must satisfy the condition
  (16) $$\frac{x_{p}^{2}}{a^{2}}+\frac{y_{p}^{2}}{b^{2}}-1\leq 0$$
2. Once inclination and rotation angles are defined, the final probe sensor *k*-th rear tip position, $P_{k}=\left[x_{k},y_{k},z_{k}\right]$ (k=2,3,4), is obtained by means of a general homogeneous transformation matrix *H*:
  (17) $$H\left(x_{p},y_{p},z_{p},\varphi_{p,x},\varphi_{p,y},\varphi_{p,z}\right)=R_{1}\times R_{2}\times R_{3}\times T$$
  where the three rotation matrices are defined as
  (18) $$R_{1}\left(\varphi_{p,x}\right)=\left[\begin{array}{cccc} 1 & 0 & 0 & 0\\ 0 & \cos(\varphi_{p,x}) & -\sin(\varphi_{p,x}) & 0\\ 0 & \sin(\varphi_{p,x}) & \cos(\varphi_{p,x}) & 0\\ 0 & 0 & 0 & 1 \end{array}\right]$$
  (19) $$R_{2}\left(\varphi_{p,y}\right)=\left[\begin{array}{cccc} \cos(\varphi_{p,y}) & 0 & \sin(\varphi_{p,y}) & 0\\ 0 & 1 & 0 & 0\\ -\sin(\varphi_{p,y}) & 0 & \cos(\varphi_{p,y}) & 0\\ 0 & 0 & 0 & 1 \end{array}\right]$$
  (20) $$R_{3}\left(\varphi_{p,z}\right)=\left[\begin{array}{cccc} \cos(\varphi_{p,z}) & \sin(\varphi_{p,z}) & 0 & 0\\ -\sin(\varphi_{p,z}) & \cos(\varphi_{p,z}) & 0 & 0\\ 0 & 0 & 1 & 0\\ 0 & 0 & 0 & 1 \end{array}\right]$$
  and the translation matrix is defined as
  (21) $$\begin{array}{c} T\left(x_{p},y_{p},z_{p}\right)=\left[\begin{array}{cccc} 1 & 0 & 0 & x_{p}\\ 0 & 1 & 0 & y_{p}\\ 0 & 0 & 1 & z_{p}\\ 0 & 0 & 0 & 1 \end{array}\right] \end{array}$$
  Therefore, the final rear sensor position will be
  (22) $$\begin{array}{c} P_{k\;[1\times 3]}=\left[P_{0k}\;\;1\right]\times H\left(x_{p},y_{p},z_{p},\varphi_{p,x},\varphi_{p,y},\varphi_{p,z}\right) \end{array}$$
  Note that only the first three elements of $P_{k}$ account for its spatial location definition. Figure 9 illustrates this procedure to re-orient the probe sensor tips over an arbitrary ellipsoid surface based on $x_{p}$ and $y_{p}$ inputs, in this case for $\varphi_{p,max}=30^{{}^{\circ}}$ and 20 sample locations (iterations).
3. Assuming a constant bubble velocity during the bubble–probe interaction, the sensor-tip trajectories are aligned with the *Z*-axis. If we define the point where the *k*-th tip contacts the bubble surface as $P_{k}^{*}=\left[x_{k}^{*},y_{k}^{*},z_{k}^{*}\right]$, the four time delays in Φ follow from:
  (23) $$P_{k}^{*}=P_{k}-V_{b}t_{k}.$$
  Recalling Equation (14), the contact times are
  (24) $$\begin{array}{c} t_{k}=\frac{z_{p}\pm c\sqrt{\Delta_{k}}}{V_{b}},\;\Delta_{k}=1-\frac{x_{p}^{2}}{a^{2}}-\frac{y_{p}^{2}}{b^{2}}, \end{array}$$
  where the ± sign corresponds to the two possible intersections of the tip trajectory with the ellipsoidal surface (entry and exit).

Equation (24) can have zero, one, or two real roots. Two real roots indicate that the rear tip crosses the bubble twice, at the upper (z>0) and lower (z<0) interfaces. A double root ($\Delta_{k}=0$) corresponds to a tangential contact at the edge (z=0), whereas a single positive root appears when the rear tip is initially inside the bubble and only the lower-surface intersection is physically relevant.

Only interactions with two real solutions will be taken into account, as it means that each tip intersects the bubble surface two times. We will label the *n*-th interaction event as a “computed bubble” event in this case and as a “missed bubble” event otherwise. If, after applying transformation *H* to a rear sensor tip, it is defined inside the bubble volume (one real solution for Equation (24)), this means that the first contact is made by a rear sensor instead by the front sensor. We will treat this case as a “missed bubble” event, as long as this event is not generally considered in real four-sensor probe signal processing.

**Figure 10:**
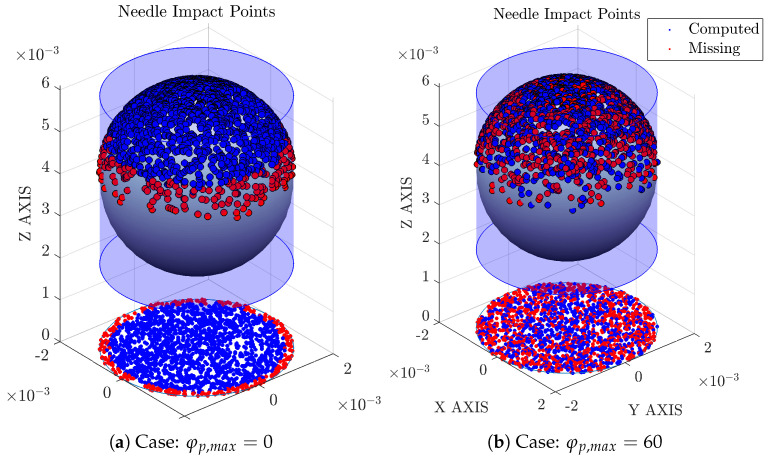
Probe inclination variability effect over computed bubble probability.

Figure 10 illustrates the mentioned steps for *n* bubble–probe interactions and how $\varphi_{p,max}>0$ (or positive radial velocity fluctuation) affects the bubble missing ratio, $m_{r}$, defined as:

(25) $$m_{r}=1-\frac{N_{bc}}{N_{b}}$$

where $N_{bc}$ is the total number of computed bubbles.

For lower inclination angles, missing bubbles are caused mainly by probe tip radial separation $r_{p}$ (interaction events near the bubble edge are not computed because one or more rear tips do not hit the bubble surface) although for higher radial velocity fluctuation, missing bubbles are also affected by the axial separation ($a_{p}$).

**Figure 11:**
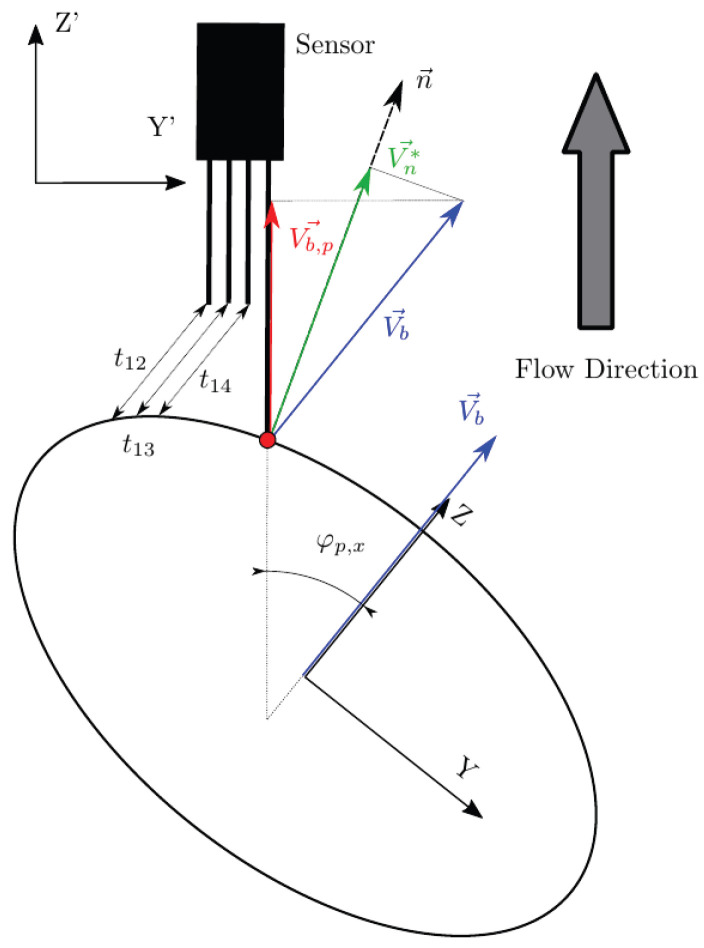
Velocities depicted from the point of view of the sensor probe.

To sum up, this framework is based on several assumptions and presents several advantages over the previous ones:

- We can consider spherical, ellipsoidal or mixed bubble geometries, thus considering a general bubble population. This could be very important for a proper sensitivity study of measurable local flow parameters: arbitrary bubble geometry definition permits us to achieve major variability in terms of bubble local curvature and chord length compared to the limited case of spherical bubble definition. It would serve to generalize obtained results, especially those measurements which are very sensitive to local curvature such as velocity, interfacial area, and chord length.
- Previous studies [6,13,14,16] were performed considering a spherical bubble geometry assumption. In this case, bubble–probe relative orientation is not important as long as all possible orientations can be achieved only by changing bubble velocity vector direction. However, if ellipsoidal bubble geometry definition is considered, bubble–probe orientation is very important to simulate more realistic and general scenarios. For example, the individual bubble–probe interaction depicted in Figure 11 can only be achieved by defining the probe location and orientation in a independent reference coordinate system.
- Considering bubble and probe coordinate systems separately provides several benefits. It permits us to define bubble geometry in a “frozen” XYZ bubble coordinate system where bubble velocity is always defined in the Z direction. It allows us to compute the individual bubble interfacial area by means of its volumetric definition (Equation (4)), since we are able to inscribe the bubble surface in a cylindrical volume always oriented in the actual bubble velocity direction, as depicted in Figure 10. It is important for setting up convergence criteria based in the interfacial area, as will be explained in Section 4.4.
- Bubble centroid velocity and interfacial velocity are defined as uncorrelated. Exact and measurable values for interfacial velocity at the bubble–probe contact point are obtained from the individual bubble shape, probe orientation, and bubble centroid velocity modulus. It allows us to perform the evaluation of measured bubble velocity and chord length without any previous assumptions and independently of local interfacial area measurement.

However, it also has limitations, since some inevitable assumptions should be made:

- The bubble velocity is constant during the probe measurement. The existing bubble velocity decrement caused by the interaction with the sensors [4,35] is neglected.
- Bubble surface is assumed to be non-deformable.

Therefore, time-dependent bubble deformation during the probe–bubble interaction is not considered. Instead, bubble shapes are represented by static spheres or ellipsoids characterized by prescribed semi-axes. This assumption is motivated by two reasons. First, in the dispersed bubbly regime and for the bubble sizes considered here, the capillary number and Weber number remain moderate, so that the instantaneous shapes observed experimentally are well represented by nearly ellipsoidal geometries. Second, our goal is to isolate the purely geometrical and kinematic contributions to the measurement bias arising from probe geometry and bubble incidence without mixing them with additional complexities due to dynamic shape oscillations. Including fully time-resolved bubble deformation would require a detailed interface-resolved simulation and would obscure the interpretation of the Monte Carlo results. Therefore, the present corrections and design guidelines should be interpreted as pertaining to flows where bubble deformation is not extreme, and where the instantaneous shape can be approximated by a slowly varying ellipsoid over the scale of the probe. Similarly, the centroidal bubble velocity is taken as constant over the duration of each individual probe–bubble interaction. In real turbulent flows, bubbles experience velocity fluctuations along their trajectory; however, over the relatively short time interval associated with a single crossing of the probe, the change in centroidal velocity is typically modest compared to the mean slip. From the viewpoint of the probe, what matters is the relative motion between the interface and the sensing points during this short interaction. Modeling each event with a constant local velocity is thus a reasonable first approximation, which allows us to clearly separate the effects of incidence angle and probe geometry from the additional scatter induced by turbulence. The impact of larger-scale turbulent accelerations is expected to manifest primarily as an increased dispersion in the measured velocities rather than as a systematic bias, and could be incorporated in future work by superimposing time-varying velocity fields onto the present Monte Carlo framework. Finally, probe rotation is used in the Monte Carlo model as a kinematic approach to represent radial velocity fluctuations and non-axial bubble trajectories. In the bubble-fixed frame used here, the bubble is held stationary while the probe translates and rotates. A non-zero radial velocity component in the laboratory frame is equivalent to the probe crossing the bubble with a tilted trajectory. Therefore, we associate the distribution of incidence angles and rotating the probe according to its kinematic equivalent, from a measurement standpoint, with bubbles approaching the probe with fluctuating radial velocity components. This representation allows us to systematically explore the effect of flow anisotropy and trajectory deviations on the missing ratio and on the reconstructed velocities and chord lengths.

## 4. Available Two-Phase Flow Parameters from Four-Sensor Probes

Since bubble geometry is defined, the present framework allows us to numerically evaluate the local flow parameters from two points of view: bubble and probe reference coordinate systems. As stated before, the velocity of the disperse phase can be computed by means of different approaches and related to the bubble itself or to its interface. In the present section we present several discussions and definitions to determine the correct velocity interpretation/measurement and its implications on the chord length measurement accuracy. In addition, we discuss the available interfacial area information from simulations and how it is used to ensure convergence for simulated cases.

### 4.1. Bubble Velocity from Simulations

The sensor probe rotates around a fixed XYZ bubble coordinate system, meaning that the sensor probe axis is not necessarily aligned with bubble centroid velocity, as depicted in Figure 11.

**Figure 12:**
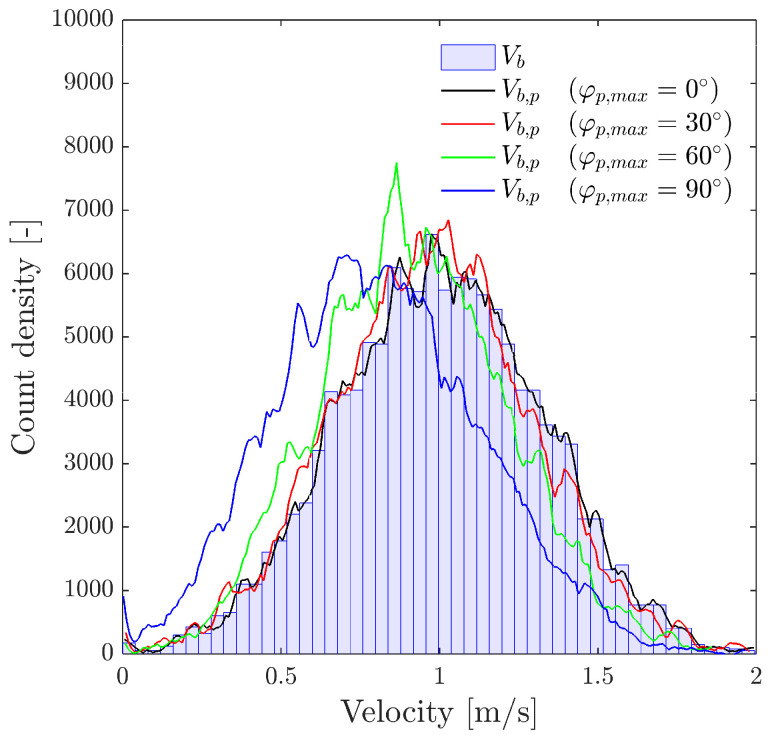
Imposed velocity $V_{b}$ from the point of view of the sensor probe, $V_{b,p}$. Changes in $V_{b,p}$ caused by the sensor probe re-orientation. In this case, using a Bivariant Gaussian distribution to define $\varphi_{p,x}$ and $\varphi_{p,y}$.

From the point of view of the sensor probe coordinate system X′Y′Z′, we can define three observable velocities with reference to the bubble–sensor contact point:

- $V_{b}$: as defined previously, it refers to bubble centroid velocity, defined in the bubble coordinate system and always in the Z direction.
- $V_{b,p}$: the bubble centroid velocity projection over the actual sensor probe axis. It is obtained by applying double homogeneous rotation $H_{V}\left(\varphi_{p,x},\varphi_{p,y}\right)=R_{1}{\left(\varphi_{p,x}\right)}_{\left[3\;\times \;3\right]}R_{2}{\left(\varphi_{p,y}\right)}_{\left[3\;\times \;3\right]}$ over $\vec{V_{b}}$ and a dot product over the Z-axis unitary vector ($\vec{u_{z}}$):
  (26) $$\begin{array}{c} \vec{V_{b,p}}=\left(\vec{V_{b}}H_{V}\left(\varphi_{p,x},\varphi_{p,y}\right)\right)\cdot \frac{\vec{u_{z}}}{|\vec{u_{z}}|} \end{array}$$
  The correct evaluation of $V_{b,p}$ from probe sensors is especially important in real measurements. Usually, in pipe flow measurements the sensor is aligned with pipe (flow) direction. In this situation, $V_{b,p}$ corresponds to local flux velocity to be integrated with local void fraction for gas flow-rate measurement and is therefore compared against the flow-meter reading [7,26,27,36,37]. In this sense, it is important to remark that $\vec{V_{b,p}}$ depends on the bubble radial fluctuation, defined by $\varphi_{p,max}$. As illustrated in Figure 12, given a $|\vec{V_{b}}|$ probability density function (PDF), the corresponding velocity PDF in the probe axis projection, velocity observed from the point of view of the virtual probe sensor, is underestimated as $\varphi_{p,max}$ increases. Therefore, both velocities, $V_{b}$ and $V_{b,p}$, should be evaluated separately because of their differing significance.
- $V_{n}^{*}$: exact interfacial velocity obtained as the projection of $V_{b}$ over the normal to surface vector $\vec{n}$ at the contact point. The normal to surface vector is obtained analytically from the surface equation and the bubble–sensor contact point individual locations.

According to definitions given in Section 2.1.3, the four-sensor probes are capable of measuring the following velocities:

- $V_{z}$: bubble velocity measured by the sensor probe in its axis direction by means of $a_{p}$, $t_{12}$, $t_{13}$, and $t_{14}$, as in Equation (12). This velocity measurement should be comparable to $V_{b}$ and $V_{b,p}$ by some extent.
- $V_{n}$: interfacial velocity measured according to Equation (13) by means of probe-obtained time delays and geometry. In contrast to $V_{n}^{*}$, $V_{n}$ accounts for probe geometry, and therefore includes errors caused by local curvature. Ideally, $V_{n}$ will provide the same value as $V_{n}^{*}$ for narrow $r_{p}$ distances and if radial bubble velocity fluctuation is neglected.

**Figure 13:**
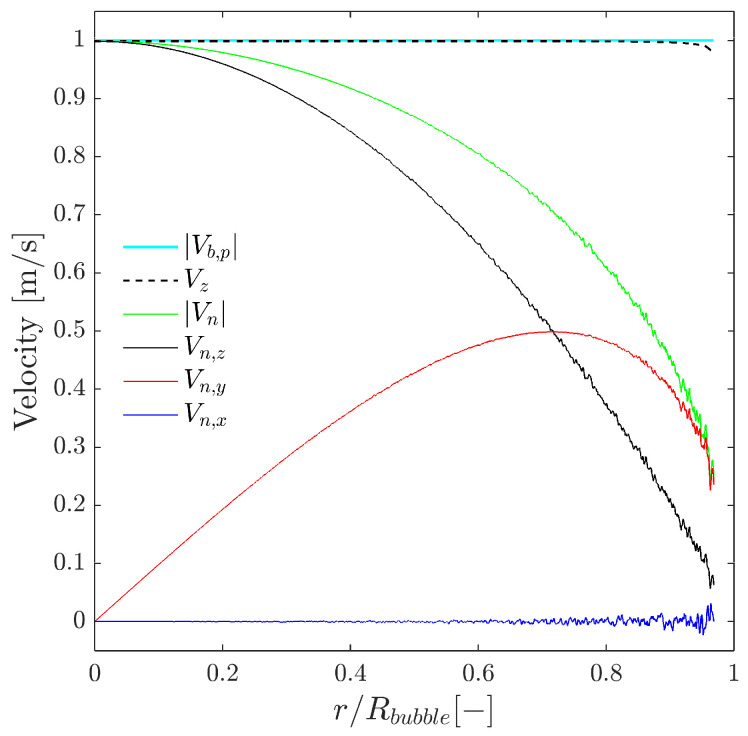
Measurable velocities obtained by a four-sensor probe along a spherical bubble radius for $r_{p}/D=0.025$. The axial velocity $V_{z}$ remains very close to the imposed bubble velocity $V_{b}$ over most of the radius, while the measured interfacial velocity $\left|V_{n}\right|$ and its axial projection $\left|V_{n,z}\right|$ decrease as the local curvature increases, leading to a strong underestimation near the bubble edge. Sampling points do not reach $r/R_{bubble}=1$ because of the finite tip separation.

As a first step, we will relate the velocities measurable by means of four-sensor probes to the physical significance of available velocities. In order to compare and relate bubble velocity to measurable bubble velocities, we will consider a simplified case as depicted in Figure 3, where $V_{b}$ is defined as unitary and parallel to the sensor probe axis (thus, $V_{b}=V_{b,p}$) and without radial bubble velocity fluctuation generated by probe-sensor rotation ($\varphi_{p,x}=\varphi_{p,y}=0$). Computing probe sensor measurable velocities along a spherical bubble radius, $R_{bubble}$, we obtain the measurable velocity profile depicted in Figure 13.

In this case, the best real bubble centroid velocity estimator available corresponds to $V_{z}$ measurement. The $|\vec{V_{n}}|$ measured value decays as bubble curvature increases (minimum near the bubble edge). This fact corroborates that neither $V_{n}$ nor its probe axis projection component, $V_{n,z}$, can be used to estimate bubble velocity $V_{b}$. Note that in Figure 13 sampling points cannot reach $r/R_{bubble}=1$, due to the radial finite separation of probe tips (in this case $r_{p}/D=0.025$). Also, the small ripple observed in all the velocity profiles except for $V_{z}$ is caused by considering $\varphi_{p,z}\neq 0$.

**Figure 14:**
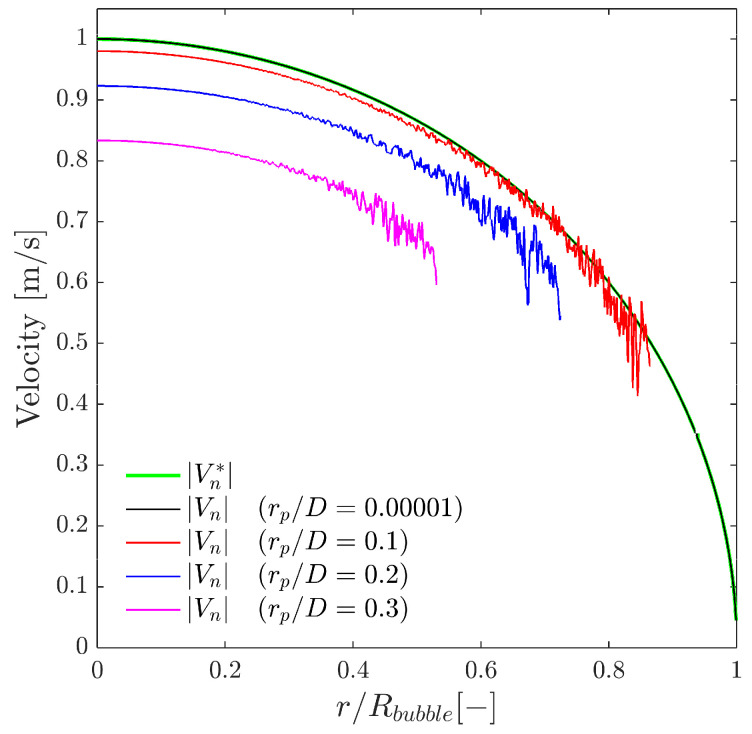
Effect of bubble curvature, expressed through the $r_{p}/D$ ratio, on interfacial velocity measurements. As $r_{p}/D$ increases, the measured $\left|\;V_{n}\right|$ increasingly underestimates the exact interfacial velocity $\left|\;V_{n}^{*}\right|$ obtained from the analytical surface normal, especially away from the bubble centre. This indicates that accurate interfacial-area estimation requires small measuring areas (low $r_{p}/D$) to keep curvature-induced bias under control.

Given the same scenario as depicted in Figure 13, Figure 14 shows the local curvature effect over the interfacial velocity measurements. As stated before, Equation (13) assumes negligible local curvature in the probe–bubble surface contact point considering the surface as a tangent plane. We can quantitatively account for an increasing local curvature by increasing the $r_{p}/D$ ratio and evaluate its influence in Equation (13). The exact value of the interfacial velocity computed analytically ($V_{n}^{*}$) is only obtained for very low $r_{p}/D$ ratios; otherwise, $V_{n}$ measured by means of Equation (13) tends to underestimate its exact value. This simple fact highlights the importance of considering the sensor probe geometry with regard to the proper local interfacial area measurement.

As a second step, we have considered more general scenarios compared to previous ones, where the bubble–sensor impact points are randomly distributed in the bubble surface instead in a single radius (similar cases as illustrated in Figure 9 and Figure 10), where

- Variability of bubble velocity is simulated by varying $V_{b}$, obtained for each bubble–probe interaction from a Gaussian distribution with mean equal to one and standard deviation of 0.1.
- Bubble radial velocity fluctuation is simulated by varying probe inclination angles, obtained from a Bivariant Gaussian distribution defined by $\varphi_{p,max}$ for each interaction event.
- Considering a spherical bubble shape, the $r_{p}/D$ ratio is set again to 0.025 in order to minimize the influence of local curvature for measurements. The $a_{p}/D$ ratio is set arbitrarily to 0.1 and we have considered spherical geometry for bubbles for 10,000 bubble–probe interactions.

**Figure 15:**
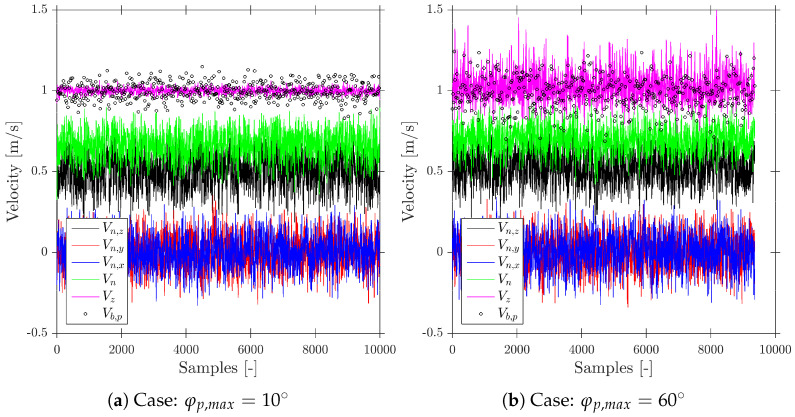
Velocity measurements for bubble–probe impact points distributed over the spherical surface, including bubble radial velocity fluctuation through a Bivariate Gaussian distribution of incidence angles. (**a**) Case with weak radial fluctuation ($\varphi_{p,max}=10^{{}^{\circ}}$); (**b**) case with strong fluctuation ($\varphi_{p,max}=60^{{}^{\circ}}$). In both cases, the axial velocity $V_{z}$ remains the best estimator of the projected bubble velocity $V_{b,p}$, whereas $\left|V_{n}\right|$ systematically underestimates $V_{b,p}$. Increasing $\varphi_{p,max}$ reduces the number of computed bubbles and broadens the velocity distributions, illustrating the impact of radial fluctuations on both missing ratio and measurement scatter.

Figure 15 depicts these two different scenarios with increasing radial velocity fluctuation variability, $\varphi_{p,max}=10^{{}^{\circ}}$ and $\varphi_{p,max}=60^{{}^{\circ}}$.

With the target value being the bubble velocity in the probe axis direction, $V_{b,p}$, we can still observe that $V_{z}$ is the best available estimator, despite the bubble radial velocity fluctuation. As expected from the previous case results, $|\vec{V_{n}}|$ tends to underestimate $V_{b,p}$. Figure 15b shows that computed bubbles are clearly less than 10,000, only caused by considering the increased variability in the probe orientation compared to the case depicted in Figure 15a.

These preliminary velocity evaluations suggests that both probe main dimensions, $a_{p}$ and $r_{p}$, are very important for velocity measurements, as stated previously in [6,13,14].

In Section 5 we will generalize the previous case by considering the probe dimensions and bubble radial velocity fluctuation intensity as random variables, hence providing generalized sensitivity maps for velocity measurements.

### 4.2. Chord Length from Simulations

Based on Figure 4, the following statement is straightforward but extremely important for practical applications: real chord length can only be correctly determined if the probe axis and $V_{b}$ are parallel. Otherwise, the chord length measuring time ($t_{0,i}$) is not linked with the measured velocity. From the point of view of numerical simulations, only for cases where $V_{b,p}=V_{b}$ (equivalent to $\varphi_{p,x}=\varphi_{p,y}=0$) will the the real chord length be recovered if $V_{z}$ is used. If $V_{n}$ is used instead, even considering $V_{b}$, parallel to probe axis, chord length will only be properly recovered if the sensor probe pierces the bubble in its center and the curvature effect is neglected (only possible by considering an ideal local probe sensor with $r_{p}/D\approx 0$).

According to the definition of the different velocity measurements from simulations, we can define three available methods for chord length (CL) calculation:

- $CL_{V_{z}}$: chord length measured by means of $V_{z}$ for each computed bubble.
- $CL_{V_{n}}$: chord length measured by means of $V_{n}$ for each computed bubble.
- $CL_{th}$: the ideal chord length, computed for all simulated bubbles ($N_{b}$) as the product of $V_{b}$ and the flight time of the front sensor tip inside the *i*-th bubble volume ($t_{0,i}$). Unfortunately, $CL_{th}$ cannot be obtained from real sensor probes. An exact value for $V_{b}$ is not available from probe measurements and cannot be computed for the whole bubble population, caused by unavoidable $m_{r}$ in real experiments.
- $CL_{th}^{N_{bc}}$: as $CL_{th}$ is the ideally measured chord length, but in this case, limited to the computed bubble population. In general, $CL_{th}^{N_{bc}}\leq CL_{th}$ cause the missed chord lengths to correspond mostly to bubbles caught near its edge (smaller chord lengths).

Therefore, there is no way to ensure the proper evaluation of the $CL_{th}$ PDF from sensor probe data. However, given $CL_{th}$ from the synthetic data, we are able to study and evaluate different probe geometry ranges where the $CL_{th}$ estimation error could be minimized (Section 7.4).

### 4.3. Interfacial Area from Simulations

Given the presented framework, we are able to compute the local time-averaged interfacial area, $a_{i}$, by multiple definitions. For *j*-th simulation iteration, based only on the information of *j*-th surface definition and *j*-th bubble–probe impact point location, $a_{i}$ can be computed given the following definitions:

- $a_{i,th}$: the present framework allows us to define a volume enclosing each bubble, which is necessary to obtain the volumetric interfacial area by means of Equation (4). It is computed as the averaged value of bubble area–volume ratios:
  (27) $$a_{i,th}=\frac{1}{N_{b}}\sum_{j=1}^{N_{b}}\frac{\Gamma_{j}}{V_{j}}$$
  where *T* is the time interval and volume *V* is defined as the cylinder enclosing the *j*-th bubble, as depicted in Figure 10. For spherical bubbles and a monodisperse bubble population, $a_{i,th}$ is a constant value because $\Gamma /V=2/R_{bubble}$.
- $a_{i,th}^{*}$: obtained by means of Equation (3), where the normal to surface vector $\vec{n}$ is computed analytically given the bubble–probe impact point P(x,y,z). In other words, it is the value for $a_{i}$ computed by means of $V_{n}^{*}$. The $a_{i,th}^{*}$ represents the ideally measured time-averaged local interfacial area, as long as it considers all bubbles simulated:
  (28) $$a_{i,th}^{*}=\frac{2}{T}\sum_{j=1}^{N_{b}}\frac{1}{|\vec{V_{n,j}^{*}}|}$$

With the data provided by the four-sensor probes, we are able to compute the local interfacial concentration by two methods:

- $a_{i,m}$: interfacial area measured by the virtual sensor probe by means of $|\vec{V_{n}}|$ as in Equation (9). Its computation requires the three time delays from rear sensors and probe geometry definition. Hence, it does not take into account missing bubbles, as information from the rear sensors is necessary. Therefore, considering only the computed bubbles, $N_{bc}$, and the front bubble interfaces:
  (29) $$a_{i,m}=\frac{2}{T}\sum_{j=1}^{N_{bc}}\frac{1}{|\vec{V_{n,j}}|}$$
  This is the equivalent measurement as if it was performed with a real sensor probe as long as it considers the deviations caused by local bubble curvature ($r_{p}/D>0$) and cannot account for missing bubbles’ interfacial area contribution.
- $a_{i,mc}$: is the corrected value for $a_{i,m}$, as long as it is obtained from $|\vec{V_{n}^{*}}|$, but only for computed bubbles:
  (30) $$a_{i,mc}=\frac{2}{T}\sum_{j=1}^{N_{bc}}\frac{1}{|\vec{V_{n,j}^{*}}|}$$
  Hence, the $a_{i,mc}$ value gives the time-averaged local interfacial area measured by an ideal sensor probe (negligible $r_{p}$, and thus, negligible error caused by local curvature) but considering the fact that bubbles are missed because of sensor probe dimensions and radial bubble velocity fluctuations. Therefore, $a_{i,mc}$ provides the exact local value for interfacial area as $a_{i,th}^{*}$, but without considering the missing bubble contribution.

On one hand, the comparison between $a_{i,th}$ and $a_{i,th}^{*}$ provides information about the available interfacial area from two points of view: volumetric and local definition. This comparison will be used to evaluate if the number of defined local bubble–probe impact points are statistically sufficient to recover the volumetric value of interfacial area concentration. On the other hand, the comparison between $a_{i,m}$ and $a_{i,mc}$ will be useful to evaluate the errors due to the finite dimensions of the sensor probe and bubble local curvature. Note that the ratio $a_{i,m}/a_{i,mc}$ is equivalent to evaluate $V_{n}/V_{n}^{*}$, therefore it also provides an insight about the error due to consider the bubble surface at bubble–probe impact point as a tangent plane [18] in the interfacial area measurement.

### 4.4. Convergence Criteria for Simulations

**Figure 16:**
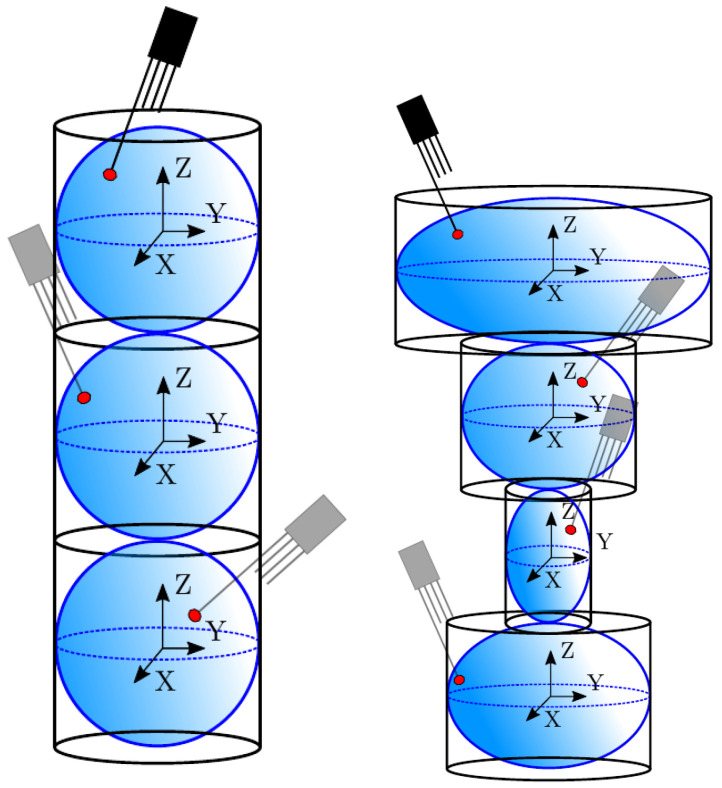
Examples of monodisperse (**left**) and polydisperse (**right**) bubble generation.

A convergence criterion based on interfacial area concentration definition would be representative, since it is a cumulative variable obtained with multiple sources of variability such as probe orientation, surface contact point, and bubble-imposed velocity. This convergence criterion is especially useful as a convergence test if we consider a polydisperse scenario, as depicted in Figure 16.

Recalling Equation (6), $a_{i,th}^{*}$ and $a_{i,th}$ should converge to the same value despite the $m_{r}$ value, as long as both measurements are computed analytically for all bubbles. If we consider a large enough number of simulated bubbles ($N_{b}$), we can rewrite Equation (6) as:

(31) $$\lim_{N_{b}\to \infty }\left(\frac{1}{N_{b}}\sum_{j=1}^{N_{b}}\frac{\Gamma_{i}}{V_{i}}\right)\equiv \lim_{N_{b}\to \infty }\left(\frac{2}{T}\sum_{j=1}^{N_{b}}{\left(a_{i,th}^{*}\right)}_{j}\right)$$

Figure 16 depicts two different cases from the point of view of bubble population generation: monodisperse (constant-radius spherical bubble shape) and polydisperse (randomly defined scalene ellipsoidal bubble shape) cases.

Considering the simple relation between sphere volume and area ratio, or the monodisperse spherical bubble generation, Equation (31) could be written as:

(32) $$\frac{2}{R_{bubble}}\approx \frac{2}{T}\sum_{j=1}^{N_{b}}{\left(a_{i,th}^{*}\right)}_{j}$$

However, for the polydisperse bubble generation case, calculation becomes more complex. For each iteration, or equivalently, for each bubble surface definition, volumetric interfacial area should be computed. This second case is more complex, since the acumulated value for the local interfacial area not only depends on probe location but also on the actual bubble shape definition.

**Figure 17:**
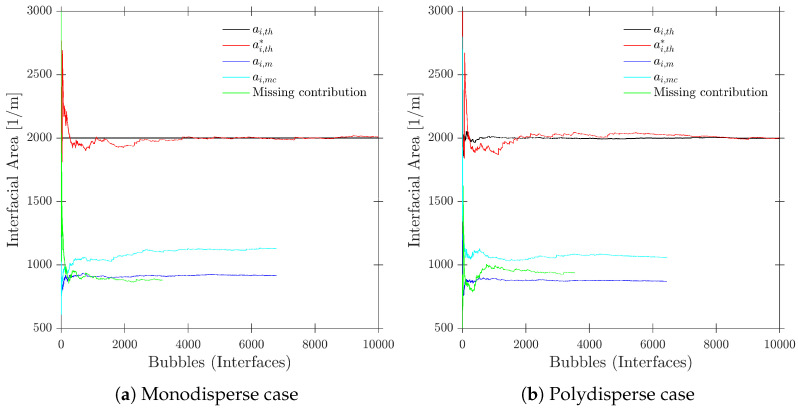
Example of convergence for polydisperse and monodisperse cases.

Figure 17 illustrates the evolution of local interfacial area with the definitions provided in the previous section and for both cases, monodisperse and polydisperse. In order to make the cases comparable, simulation parameters have been defined as follows: $\varphi_{p,max}=30^{{}^{\circ}}$ with $\varphi_{p,x}$ and $\varphi_{p,y}$ obtained from a Bivariant Gaussian PDF, same tetragonal geometry with $a_{p}/D=1$ and $r_{p}/D=0.1$, and same mean bubble diameter bubble population, D=2 mm, for both cases. From the point of view of simulation set-up, comparison between $a_{i,th}$ and $a_{i,th}$ provides the necessary information on whether the generated bubbles are sufficient in order to recover the interfacial area value from local measurements. The convergence of $a_{i,th}^{*}$ to $a_{i,th}$ will provide insight about the minimum number of iterations or generated bubbles to theoretically obtain a proper value for the measured local interfacial area. This becomes especially important in the polydisperse case, where even the volumetric interfacial area changes in time, as depicted in the earliest values for $a_{i,th}^{*}$ in Figure 17b.

In practical terms, these convergence criteria also bound the residual Monte Carlo sampling uncertainty of the reported quantities. For each simulated case, we generate $10^{4}$ bubble–probe interactions for spherical, monodisperse populations and $3\times 10^{4}$ interactions for polydisperse populations to account for the extra variability introduced (Table 2), values selected from preliminary convergence tests as the minimum sample sizes needed to reduce the discrepancy between volumetric and local interfacial-area estimates below 1%. We only retain cases for which the difference between $a_{i,th}$ and $a_{i,th}^{*}$ is below 1% (Equation (31)). Although convergence criteria are usually fulfilled before the total bubble computations, extra generated bubble information will be useful to provide more data with regard to velocity and chord length statistical evaluation. In addition, the stability requirement on $a_{i,m}$ in Equation (33) ensures that the cumulative estimate changes by less than the prescribed relative tolerance over at least 200 successive computed interfaces, which typically corresponds to about $10^{4}$ detected bubbles as observed in preliminary simulations (Figure 17). As all other observables ($V_{z}$, $V_{n}$, $a_{i,m}$, chord lengths) are evaluated over the same set of bubble–probe interactions once these criteria are satisfied, their residual statistical uncertainty due to random sampling is of the same order or smaller than for $a_{i}$. The colour maps and probability density functions shown in the following sections should therefore be interpreted as converged mean responses of the probe, where the remaining Monte Carlo noise is negligible compared with the systematic trends induced by probe geometry and incidence-angle statistics.

As stated, only once convergence is validated by means of Equation (31) can we consider the case to evaluate the stability of the sensor probe-measured local interfacial area, $a_{i,m}$. Therefore, the necessary condition for $a_{i,m}$ stable measurement of at least 200 subsequent values for $a_{i,m}$ must satisfy the following condition:

(33) 100×|ai,m−ai,mNbc|ai,mNbc≤εIm(%)

where $a_{i,m}^{N_{bc}}$ is the last computed value for $a_{i,m}$ in the simulated case and εIm is set to 1. This criterion would provide the minimum number of interfaces ($I_{m}$) or computed bubbles necessary to obtain a reliable value for measured interfacial area within an admissible interval relative error, εIm (%). This is important and useful in practical applications as it could serve to determine the minimum necessary measuring time to perform the experiments; detailed results are presented in Appendix A.

As also depicted in Figure 17, the missing bubble contribution, obtained as the difference between $a_{i,th}^{*}$ and $a_{i,mc}$ (only available from numerical simulations, as it is computed from $|\vec{V_{n}^{*}}|$), could be obtained and evaluated for further analysis.

## 5. Simulation Definition and Review of Previous Works

Numerical simulations used in this work are organized according to the three configurations defined in Table 2, each one intended to illustrate the four-sensor probe performance in real and different experimental situations by setting up the definitory parameters in the numerical framework.

The four simulation cases summarized in Table 2 are defined through the following common workflow: (i) select the probe geometry $\left(a_{p},r_{p}\right)$ and its position with respect to the wall; (ii) generate a synthetic bubble population (size, shape, and centroidal velocity) and sample incidence angles $\varphi_{p,x},\varphi_{p,y}$ from the prescribed PDFs; (iii) transform the probe into the bubble-fixed frame and compute probe–bubble intersections; (iv) classify bubbles as computed or missed and evaluate the observables ($t_{0,i}$, $V_{z}$, $V_{n}$, $a_{i,m}$, $CL_{Vz}$, $CL_{V_{n}}$, $m_{r}$); and (v) repeat until the convergence criteria on $a_{i}$ and $m_{r}$ are satisfied. The ranges of $a_{p}/D$ and $r_{p}/D$ in Table 2 are chosen to cover and slightly extend the geometry ratios reported for practical four-sensor probes in vertical bubbly flows [10,27,38,39] and also from two-sensor probes [6,13,26], so that both typical designs and more extreme configurations can be assessed within a realistic envelope.

As a first step towards the selection of the proper simulation definition parameters depicted in Table 2, we have reviewed previous works’ conclusions in order to set up a proper starting point, specially for the four-sensor probe’s dimension ranges and variability. Similar studies based on Monte Carlo simulations have been carried out. We would highlight the previous works [6,13,14] where the authors performed a similar study based on a Monte Carlo approach for double-sensor probe accuracy, considering spherical bubble assumption and isotropic bubble velocity field definition. They proposed several probe sensor geometry operating limits to be used regarding velocity and interfacial area measurement accuracy. For example, ref. [6] proposed the following probe geometrical criteria in order to minimize bubble velocity overestimation by using dual-sensor probes:

(34) $$0.5\leq a_{p}/D\leq 2$$

The reasons for setting up the lower limit were the convergence of the method and maximum 30% velocity overestimation. The upper limit was considered with regard to the feasibility of obtaining a proper corrective velocity factor.

Also considering dual-sensor probes, ref. [14] proposed a more restrictive interval for the axial ratio criteria:

(35) $$0.6\leq a_{p}/D\leq 1$$

In this study, the upper limit was set considering that bubbles reach rear sensors before leaving the front sensor in order to limit the hydrodynamic effect in the simulations. Both criteria were suggested by considering discrete values for radial velocity fluctuation and discrete values for the $r_{p}/D$ ratio, thus providing limited interaction variability with $a_{p}/D$ and $r_{p}/D$ ratios.

Taking into account these previous studies and conclusions, we have performed a global simulation (S-Wide) with wider intervals for $a_{p}/D$ and $r_{p}/D$ ratios to explore the real limitations of the four-sensor probe design and observe the possible similarities. Spherical bubble shape is imposed in S-wide to make results comparable.

**Figure 18:**
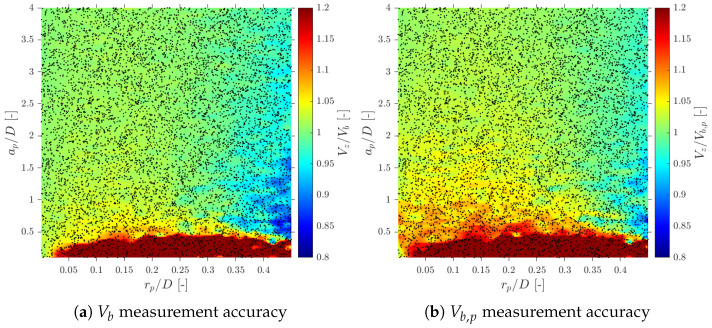
$V_{z}$ velocity measurement for axial and bubble centroid velocities.

Despite the fact that we are considering four-sensor probes instead of double-sensor probes (using Equation (11) instead of Equation (12)), we have found remarkable similarities. As depicted in Figure 18a, where each dot corresponds to a single simulated case in S-Wide simulation, a lower limit for the $a_{p}/D$ ratio greater than 0.5 is necessary to avoid overestimation of $V_{b}$, in agreement with [6,14]. Although $V_{b,p}$ is in the selected range for the $a_{p}/D$ and $r_{p}/D$ ratios, the observed measured velocity overestimation is limited to 15–20% even considering $a_{p}/D\geq 1$; therefore, we cannot set a proper upper limit for geometrical ratios only based on velocity measurements.

Le Corre and Ishii [14] also proposed a specific geometric criteria for four-sensor probe interfacial area measurement based on the $a_{p}/D$ ratio, but less restrictive than Equation (35). In this work, the authors also suggested a corrective factor for measured interfacial area. The most important achievement was that the proposed correction factor $a_{i,corr}$ (Equation (36)) was only based on the missing ratio, $m_{r}$, and in a wide interval (0 ≤ $m_{r}$ ≤ 0.7), which is a direct and easy parameter to obtain from four-sensor probes in real experiments.

(36) $$a_{i,corr}=\frac{1}{\sqrt{1-\sqrt{2.4m_{r}-1.5{m_{r}}^{2}}}}\;m_{r}\in \left[0,0.7\right]$$

**Figure 19:**
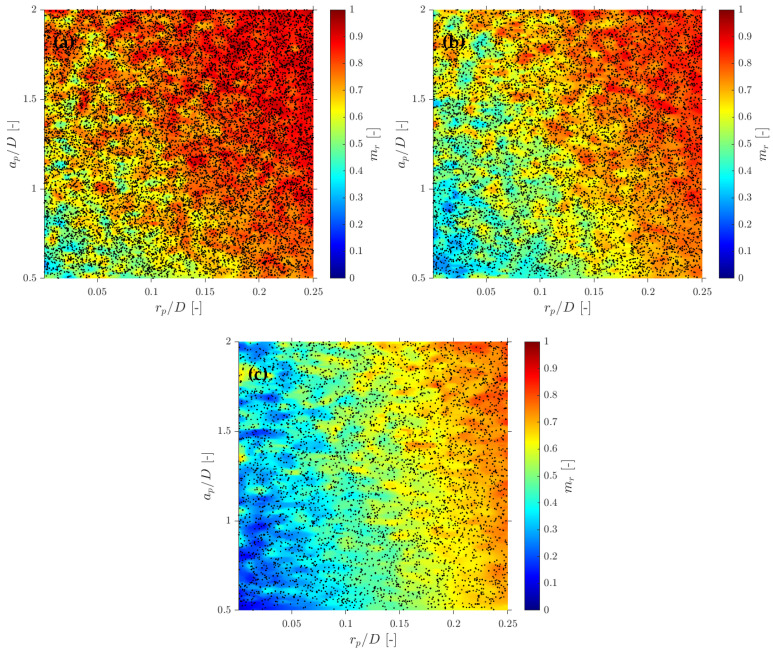
Missing–ratio maps for the S-Wide simulation, showing the evolution of the $m_{r}$ as a function of ($a_{p}/D$, $r_{p}/D$) for decreasing ranges of bubble radial–velocity fluctuation: (**a**) wide distribution of bubble–probe incidence angles, (**b**) intermediate fluctuation level and (**c**) limited fluctuation representative of upward bubbly flow. In all cases the attack angle $\varphi_{p}$ is sampled from a bivariate Gaussian PDF.

In terms of interfacial measurements, as stated by [14], the missing ratio is a good indicator to estimate the interfacial area measurement error as long as it indirectly includes the proportion of missed bubble interfacial area contribution. Therefore, $m_{r}$ would be considered as a key parameter. Moreover, low values for $m_{r}$ is an indicator that the flow condition is successfully measured as it indicates that a wide range of bubble dimensions are captured and taken into account for local parameter calculation. Thus, any probe geometrical consideration will be headed to minimize $m_{r}$. In addition to probe geometry aspects, it is also important to determine the relationship between the flow definition parameters and $m_{r}$. For example, Figure 19a shows the similar influence of the $a_{p}/D$ and $r_{p}/D$ ratios in the computed $m_{r}$ if the full range of bubble radial velocity fluctuation is considered. However, this trend changes if bubble radial velocity fluctuation is limited. Considering the case of an upward air–water bubbly flow, Serizawa et al. [40] found that the maximum observable ratio between axial and radial bubble velocity was about 0.4. From the point of view of numerical simulations, it corresponds to a maximum angle of attack $\varphi_{p,max}\leq 35^{{}^{\circ}}$. Filtering the cases from S-Wide considering a $\varphi_{p,max}$ of about $35^{{}^{\circ}}$ in order to simulate an upward bubbly flow by limiting the bubble radial velocity fluctuation, we obtain Figure 19c. As expected, as bubble radial velocity fluctuation decreases, the effect of axial distance is less important in terms of computed bubble probability; therefore, in this situation, measurement accuracy would be more dependent on the $r_{p}/D$ ratio than the $a_{p}/D$ ratio. This fact is important since it demonstrates the critical influence of bubble radial fluctuation over measurable parameters and its influence on the selection of the critical sensor probe design parameters.

**Figure 20:**
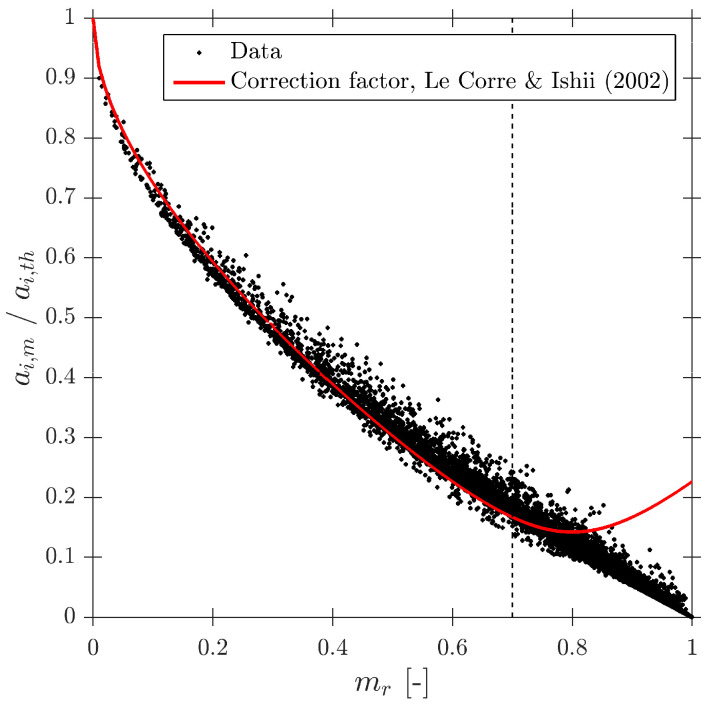
Interfacial area correction factor and data from S-Wide.

As defined in Table 2, for the S-Wide simulation we have used two different probe tip arrangements, square and tetragonal, which are defined in Section 6. The objective was to be able to compare our simulation results with the interfacial area measurement simulation data in [14]. Figure 20 illustrates the good agreement of interfacial area ratios obtained from S-Wide simulation data and the proposed correction factor by [14] with Equation (36) for four-sensor probe geometries, even considering an extended range for $a_{p}/D$ and $r_{p}/D$ ratios. This fact corroborates the accuracy and the wide range of applicability ($m_{r}\leq 0.7$) of Equation (36) despite the probe dimensions and bubble radial velocity fluctuation.

## 6. Evaluation of Probe Geometry Influence

The four-sensor probe can effectively obtain the local interfacial area data for bubbles having a large size relative to the probe. The curvature of the bubble interface at the measuring point is of utmost importance, as it determines the accuracy in the interfacial area calculation by means of the proposed methodology of [18]. The key parameter to ensure a correct measurement in terms of local curvature is the measuring area formed by the probe. If the bubbles have a similar size to the probe measuring area (defined by the radial distance of the rear tips), the error due to the curvature of the interface may be significant [41].

**Figure 21:**
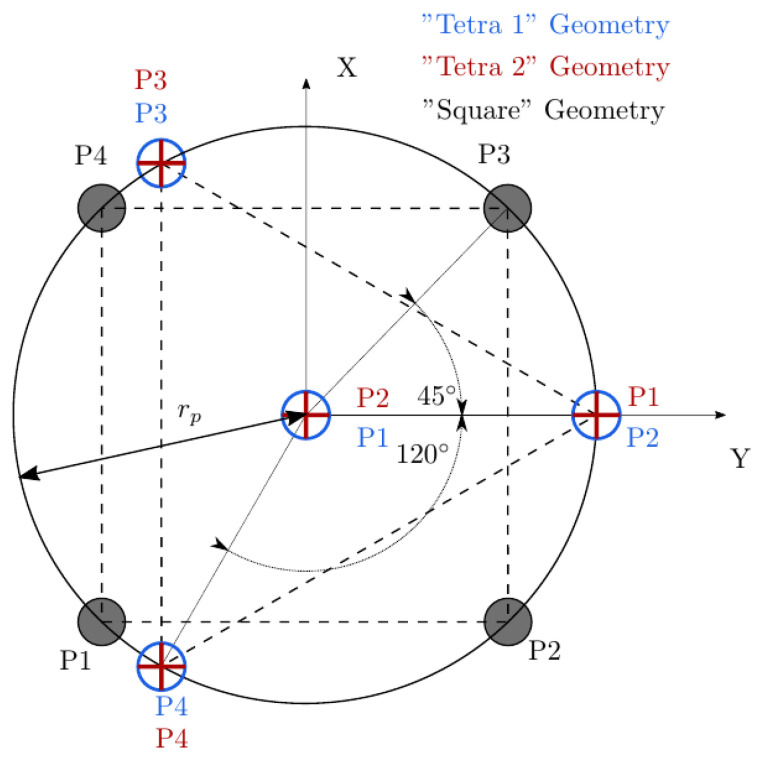
Probe geometry arrangements.

We have tested three different but common four-sensor probe geometries and evaluated their performance. In order to make the results from the different geometries comparable, the area enclosed by the axial projection of the sensor probe tips should be similar. This will ensure that the local surface curvature influence on measurable variables such as interfacial area measurement, $m_{r}$, and velocity ratios will be also comparable. The three different four-sensor probe geometries evaluated are labeled as *Square*, *Tetra1*, and *Tetra2*, geometrically defined in Table 1 and Figure 21.

**Figure 22:**
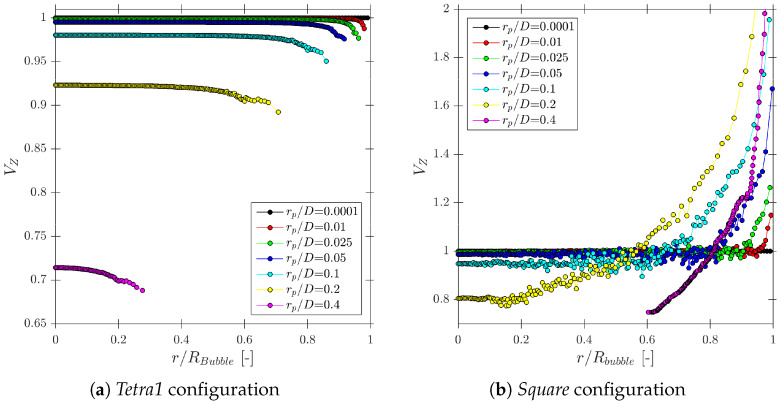
Influence of $r_{p}/D$ and probe geometry on bubble velocity measurements for the *Tetra1* and *Square* configurations. The profiles show that, for sufficiently small $r_{p}/D$, both geometries provide similar $V_{z}$ accuracy, whereas larger $r_{p}/D$ values amplify curvature effects and increase the overestimation of bubble velocity. This confirms that velocity errors are primarily governed by $r_{p}/D$, with geometry playing a secondary role once the measuring area is small.

Considering again the simplest case, where unitary $V_{b}$ is parallel to the sensor probe axis ($\varphi_{p,x}=\varphi_{p,y}=0$) and probe sensor sampling points along a spherical bubble radius with random tip orientation ($\varphi_{p,z}\in \left[0^{{}^{\circ}},360^{{}^{\circ}}\right]$), we can study the theoretical measurement accuracy for each probe geometry and only for $r_{p}$ variations. Considering “*Tetra1*” (Figure 22a) and “*Square*” (Figure 22b) geometries, we can observe several differences.

If the $r_{p}/D$ ratio is small enough, the error committed due to the bubble curvature inside the probe measuring area does not affect the bubble velocity estimation. As the $r_{p}/D$ ratio increases or measurements are performed near the bubble edge (maximum curvature), the bubble velocity estimation accuracy decreases. A substantial difference has been found depending on the probe arrangement evaluated. On one hand, for *Tetra1* velocity tends to be underestimated for all $r_{p}/D$ ratios. For the highest $r_{p}/D$ ratios close to 0.5, only bubbles caught near the center could be computed. On the other hand, for *Square*, velocity tends to be overestimated and only evaluated near the bubble edge for higher $r_{p}$ ratios. This probe arrangement dependence can be explained as follows: in *Square* geometry, if bubble is caught near the edge, the only viable measurement requires rear sensors oriented towards the bubble center, otherwise rear sensors will not contact the bubble surface. Recalling Figure 3, this condition implies that $t^{*}>t_{12},t_{13},t_{14}$, thus, according to Equation (12), an overestimated bubble velocity would be obtained. This condition does not occur if the *Tetra1* configuration is used, as long as rear sensors are equally distributed around the front sensor. With the *Tetra1* configuration, velocity underestimation is caused by an opposite condition: if $r_{p}$ distance is similar to bubble radius, viable measurements imply that the front sensor pierces the bubble near the bubble center, while rear sensors pierce the bubble near the edge; thus, $t^{*}<t_{12},t_{13},t_{14}$. These observations, even without considering radial bubble velocity fluctuation, illustrates that the probe geometry and dimensions would influence velocity measurement accuracy and are therefore important to take into account for simulations for proper four-sensor probe design criteria.

Considering more realistic simulations as in S-Geometry, which includes a polydisperse (spherical) bubble population, and Gaussian PDF for bubble centroid velocity ($V_{b}$), we evaluate the three different probe geometries’ effect on local flow measurements: velocity, interfacial area, and chord length.

### 6.1. Velocity Measurements

**Figure 23:**
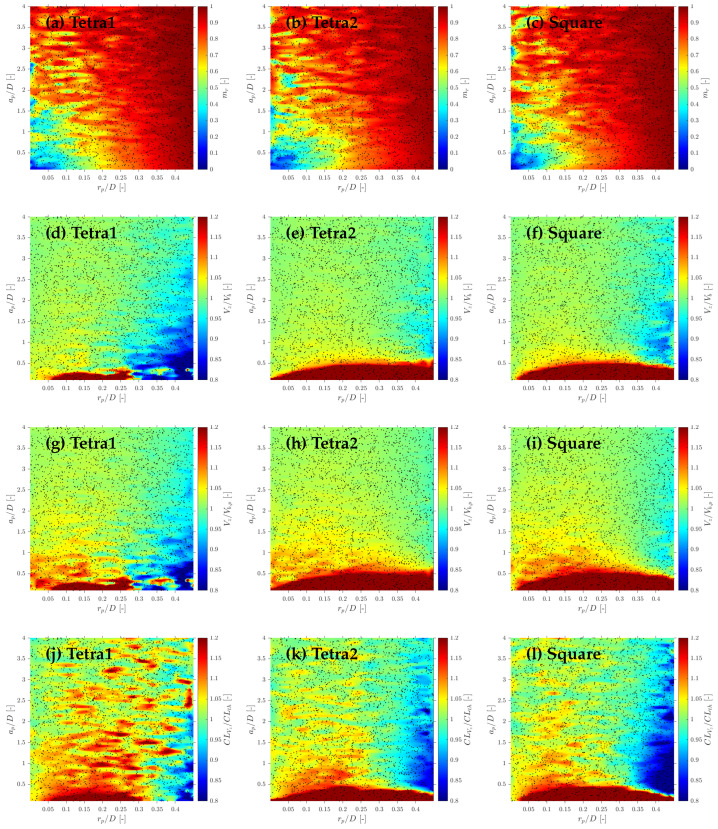
Effect of probe geometry on missing ratio and velocity- and chord length-related quantities in the S-Geom simulation. Panels (**a**–**c**) show the missing ratio $m_{r}$ for *Tetra1*, *Tetra2*, and *Square* probes; (**d**–**f**) show the flux velocity ratio $V_{z}/V_{b,p}$; (**g**–**i**) show the centroid velocity ratio $V_{z}/V_{b}$; and (**j**–**l**) show the chord length ratio $CL_{Vz}/CL_{N_{bc}}^{th}$. For all geometries, errors are weakly sensitive to $a_{p}/D$ but strongly dependent on $r_{p}/D$: within $r_{p}/D≲0.25$, the three configurations display comparable performance.

Considering the bubble radial velocity fluctuation effect, Figure 23 shows how bubble axial velocity measurement ($V_{z}$) is affected by probe geometry within a wide range for $a_{p}/D$ and $r_{p}/D$ ratios. For higher $r_{p}/D$ ratios, velocity is underestimated by the *Tetra1* configuration, while it is overestimated for the *Tetra2* and *Square* geometries for lower $a_{p}/D$ ratios. These results are consistent with Figure 22’s radial velocity profiles: considering random changes in sensor probe orientation ($\varphi_{p,max}>0$) and the specific condition of a high $r_{p}/D$ ratio, viable bubble velocity measurement is likely to be performed with priority only in those cases when the front sensor pierces the bubble near to its center, therefore obtaining an underestimated $V_{z}$. However, it can be found that velocity measurement errors are very similar within $r_{p}/D\in \left[0,0.25\right]$ range, despite the probe configuration.

Therefore, it can be concluded at this point that if the probe measuring area is sufficiently small, errors due to curvature are not significant and are not affected by probe tip configuration; otherwise, probe geometry greatly affects velocity measurements. However, even for high radial velocity fluctuations, there is an optimum geometry limit where the probe response is quite similar.

### 6.2. Interfacial Area Measurements

**Figure 24:**
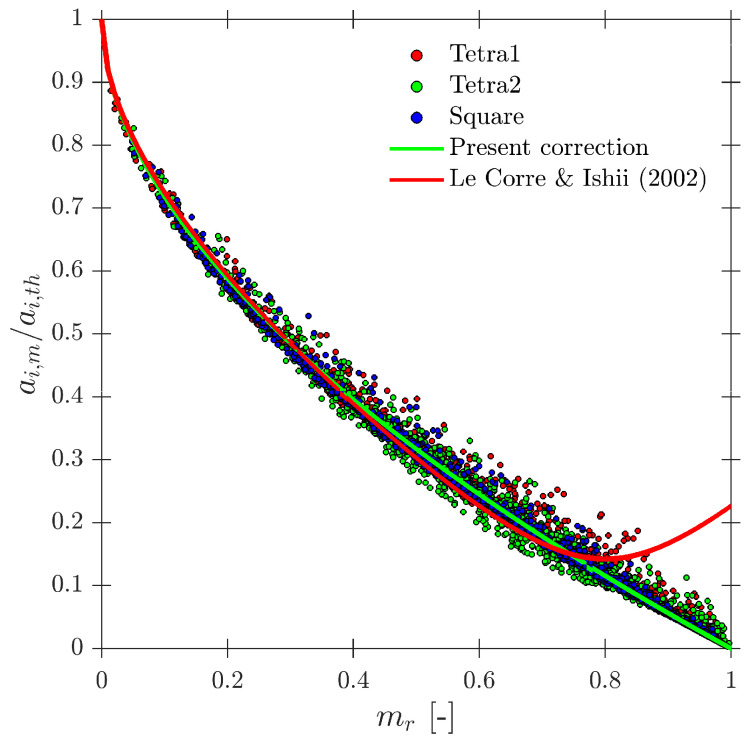
Probe geometry effect over missing ratio.

The corresponding $m_{r}$ mapping for each geometry is shown in Figure 23a–c. The probe geometry does not have an important effect on $m_{r}$ even considering the whole range for $r_{p}/D$ and $a_{p}$ ratios. This fact suggests that the applicability of the correction factor proposed by [14] is still accurate, despite the probe configuration. By plotting the interfacial area ratio ($a_{i,m}/a_{i,th}$) for tested probe geometries, as illustrated in Figure 24, it is confirmed that the interfacial area ratios for S-Geometry simulation collapse according to [14]’s prediction.

Ref. [14] used only 100 simulated cases and limited to 10,000 iterations (simulated bubble–probe interactions) in order to obtain the correlation in Equation (36). Although this number of iterations provides sufficient statistical information to evaluate interfacial area or velocity within the $m_{r}\in \left[0,0.7\right]$ range, we have found that it is insufficient beyond $m_{r}=0.7$. We have used data from S-Geom and S-Main ($1\times 10^{4}$ cases and $3\times 10^{4}$ iterations for each one) to extend and re-evaluate the correlation in a full $m_{r}$ range, providing a sufficient number of iterations to validate the convergence interfacial area measurements and also provide sufficient statistical information to compute measured velocities and chord lengths, even for higher $m_{r}$ values. The proposed correction factor (shown in Figure 24), obtained by least squares fitting, is:

(37) $$a_{i,corr}^{*}=\frac{1}{1-m_{r}^{0.55}}\;m_{r}\in \left[0,1\right]$$

In any case, we would like to remark that both correction factors (Equations (36) and (37)) present similar accuracy in the $m_{r}\in \left[0,0.7\right]$ range, providing relative errors below 6.5% in 95% confidence bounds.

**Figure 25:**
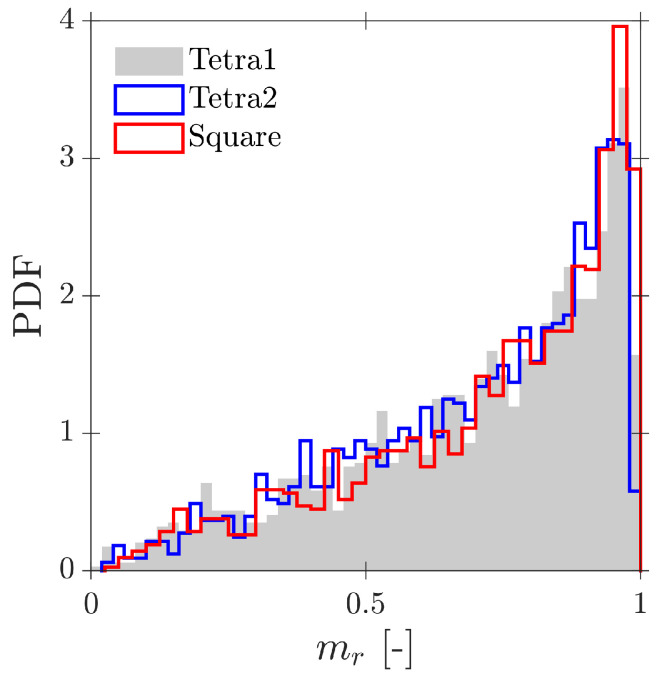
Probe geometry: bubble detection efficiency.

In [14], the authors used the effective bubble ratio (the opposite of $m_{r}$, computed as $N_{bc}/N_{b}$) to evaluate the *Square* and *Tetra1* geometries, using efficiency in order to minimize the missing bubble signal. Based on their simulation results, ref. [14] concluded that *Square* was more efficient despite probe tip radial separation or bubble radial velocity fluctuation. They considered needles separated by a radial gap *d*, causing the area enclosed by the probe tips in the *Square* configuration to be greater than in the *Tetra1* configuration. We have performed the same study but considered a similar enclosed area for all geometries, as depicted in Figure 21, and considering the obtained $m_{r}$ PDF for each geometry. As illustrated in Figure 25, no remarkable difference has been found between tested geometries in terms of bubble detection efficiency.

### 6.3. Chord Length Measurements

As illustrated in Figure 23j–l, chord length measurements are also likely to be affected by probe geometry since it is linked to bubble velocity. Although we considered that real chord length cannot be precisely recovered, it is possible to limit the range of applicability of sensor probes based on geometry limits. On one hand, even in an ideal vertical flow we are limited to computing $V_{z}$ and $V_{n}$ velocities, in principle, both smaller than $V_{b}$. Therefore (neglecting errors due to bubble curvature), $CL_{V_{n}}$ and $CL_{V_{n}}$ are expected to underestimate $CL_{r}$. However, this chord length underestimation could be offset to a certain extent by the fact that $V_{z}$ and $V_{n}$ are overestimated when local bubble curvature is taken into account. As a preliminary result, considering only computed bubbles, the $CL_{V_{z}}/CL_{th}$ ratio has been mapped in Figure 23j–l for the three geometries tested. In general, chord length tends to be overestimated except at the highest $r_{p}/D$ ratios for the *Tetra2* and *Square* geometries. Again, these results are consistent with the previous ones depicted in Figure 22: considering random changes in sensor probe orientation ($\varphi_{p,max}>0$) and the specific condition of a high $r_{p}/D$ ratio, viable chord length measurement is likely to be performed with priority only in those cases when the front sensor pierces the bubble near to its center. This causes $t^{*}<t_{12},t_{13},t_{14}$ and, therefore, an underestimation of the chord length.

As a conclusion, it is interesting to remark that changes in geometry are an important factor to be considered. Measurements are quite insensitive in the range selected for the $a_{p}/D$ ratio but are highly dependent on the $r_{p}/D$ ratio. However, considering the $V_{z}/V_{b,p}$ and $V_{z}/V_{b}$ ratios, probe performance is very similar despite probe geometry in the $r_{p}/D\in \left[0,\;0.25\right]$ range.

## 7. General Bubbly Flow Evaluation

The previously presented simulation results from S-Geom, S-Wide (Table 2), and individual cases used to depict the possible bubble velocity measurements, under spherical bubble assumption, are in accordance with previous works: the need to carefully consider the sensor probe dimensions ($a_{p}/D$, $r_{p}/D$ and geometry arrangement) and the necessary interfacial measurement correction.

S-Main simulates more general but complex scenarios compared to S-Geom and S-Wide simulations. It accounts for randomly generated ellipsoidal bubble population and increased bubble–probe interaction variability from two sources: relative bubble–probe angle distributed by uniform probability over bubble surfaces (simulating a more enhanced variability in a three-dimensional flow, compared to a B-Gaussian distribution) and variable bubble centroid velocity modulus.

Based on previous parameter sensitivity maps, we would suggest the following dimensional limits for probe design used for S-Main simulation:

(38) $$\begin{array}{c} 0.5\leq a_{p}/D\leq 2\\ 0\leq r_{p}/D\leq 0.25 \end{array}$$

**Figure 26:**
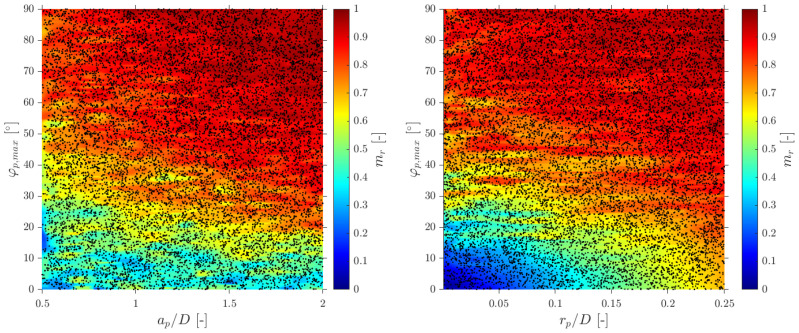
Expected $m_{r}$ considering radial buble velocity fluctuation and probe dimensions.

The upper limit for the $r_{p}/D$ ratio is suggested based on the CL, $V_{b}$, and $V_{z,b}$ evaluation shown in Figure 23d–l. The lower limit for $a_{p}/D$ is selected, as stated previously, to prevent large $V_{b}$ and $V_{b,p}$ overestimation. Under the assumption of constant $V_{b}$ in the bubble–probe interaction time, there is no evidence of an upper limit for the $a_{p}/D$ ratio based on velocity or chord length measurements. This upper limit determination has been considered from $m_{r}$ sensitivity maps (Figure 23a–c). We have considered $a_{p}/D\leq 2$ as an upper limit, as it produces a similar $m_{r}$ as $r_{p}/D\leq 0.25$. This condition is equivalent to assume the same errors produced radial velocity fluctuation in both dimensions of the sensor probe, as illustrated in Figure 26, which depicts the influence of bubble radial velocity fluctuation in terms of $\varphi_{p,max}$ over $m_{r}$.

### 7.1. Effect of Bubble Geometry (Spherical/Ellipsoidal) and Interfacial Area Measurements

**Figure 27:**
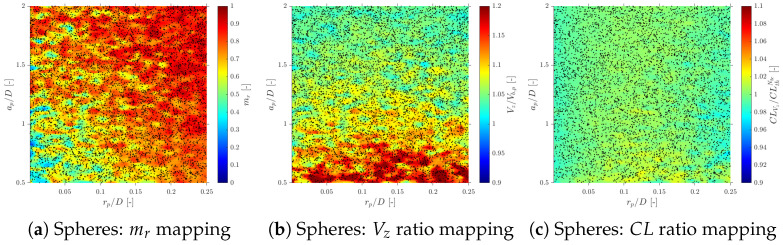
Local variable mapping for spherical bubble population.

**Figure 28:**
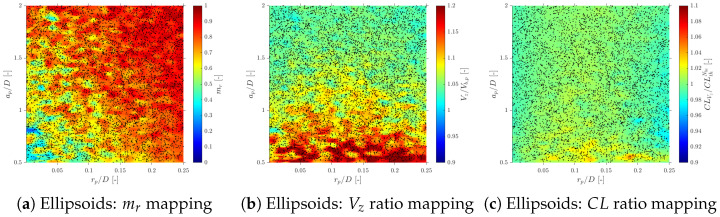
Local variable mapping for ellipsoidal bubble population.

**Figure 29:**
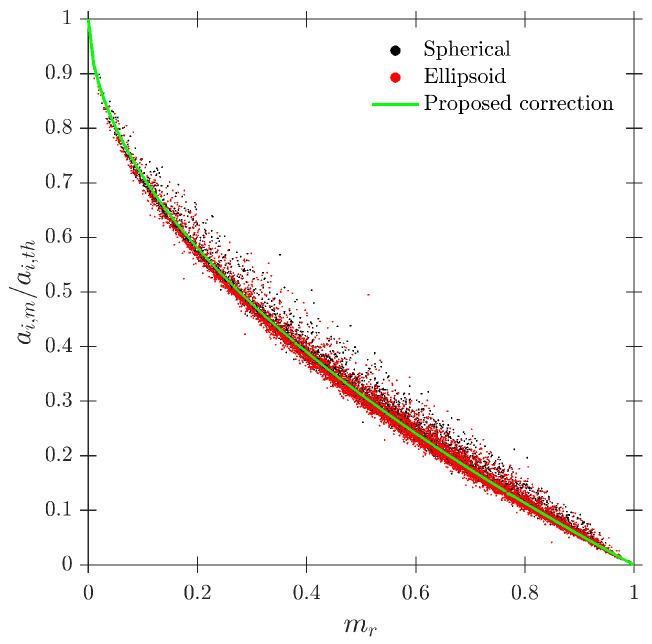
$a_{i,m}/a_{i,th}$ ratios from S-Main.

For the probe dimension ranges selected, the $m_{r}$ depicted in Figure 27a and Figure 28a presents almost the same profile distribution, which is in accordance with Figure 29. Figure 27b and Figure 28b show that the error measurement in $V_{z}$ is also similar, where significant velocity overestimation is detected in the range of $a_{p}/D\in \left[0.5,\;0.9\right]$ and the whole range for $r_{p}/D$. Outside this probe dimension interval, the maximum relative errors detected for the velocity measurements are almost uniform and under 15%. The chord length ratio for computed bubble population, depicted in Figure 27c and Figure 28c, presents similar accuracy.

The correlation for measured interfacial area correction proposed by Le Corre and Ishii [14] relies on the accurate measurement of $|\vec{V_{n}}|$, and therefore it is dependent on the probe geometry, the bubble local curvature, and, by extension, the bubble shape definition, assumed to be spherical at this point. Most of the measurable interfacial area loss comes from missing bubbles and theoretically can be correlated with $m_{r}$. However, the extra error induced by bubble curvature in the $V_{n}$ measurement accuracy cannot be neglected and should be studied for arbitrary bubble shapes to extend the applicability of this correlation.

By plotting the $a_{i,m}/a_{i,th}$ ratio against $m_{r}$ separately for ellipsoidal and spherical bubble cases for S-Main simulation (Figure 29), it can be observed that the obtained results collapse in the proposed correlation, as if spherical assumption is considered (Figure 24). Again, this fact suggests that there is no significant effect by considering ellipsoidal rather than spherical bubble definition in terms of interfacial area recovery.

### 7.2. Effect of Bubble–Probe Angle Definitory PDF

On one hand, if Bivariant Gaussian PDF is used to distribute $\varphi_{p}$ we can assume that there is a predominant bubble–probe attack angle, as in a vertical flow configuration. On the other hand, if a uniform PDF is used, we could expect a more complex scenario with different non-desirable effects:

- Increase in $m_{r}$ and major sensitivity to $a_{p}/D$ ratio.
- Higher variability and errors in $V_{b}$ and $V_{b,p}$ ratio estimation and, by extension, in $CL_{V_{z}}/CL_{th}^{N_{bc}}$ ratio estimation.

**Figure 30:**
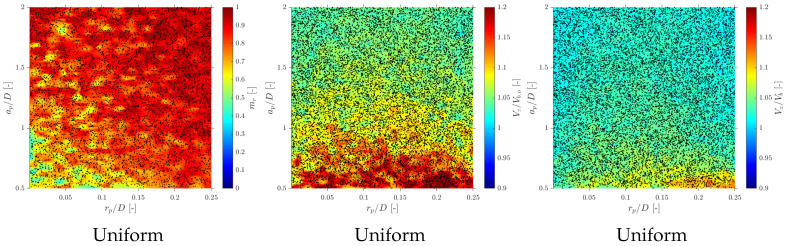

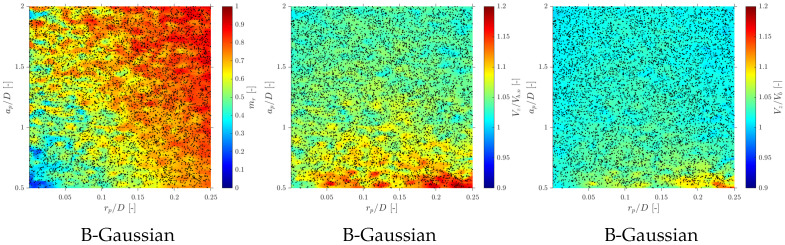
Effect of bubble–probe attack angle definitory PDF (Uniform or B-Gaussian) over $m_{r}$, $V_{z}/V_{b,p}$, and $V_{z}/V_{b}$.

Those effects, depicted in Figure 30, would be even more important if random bubble shape is considered. As expected, a uniform PDF produces a significant increase in $m_{r}$ and deviations in $V_{b,p}$. However, no significant effect is observed in $V_{b}$ estimation or $CL_{V_{z}}/CL_{th}^{N_{bc}}$ ratio. As depicted previously in Figure 29, corrected interfacial area measurements are unaffected. As stated, the main parameter affected by changing $\varphi_{p}$ PDF is the flux velocity.

### 7.3. Bubble Velocity Measurements

**Figure 31:**
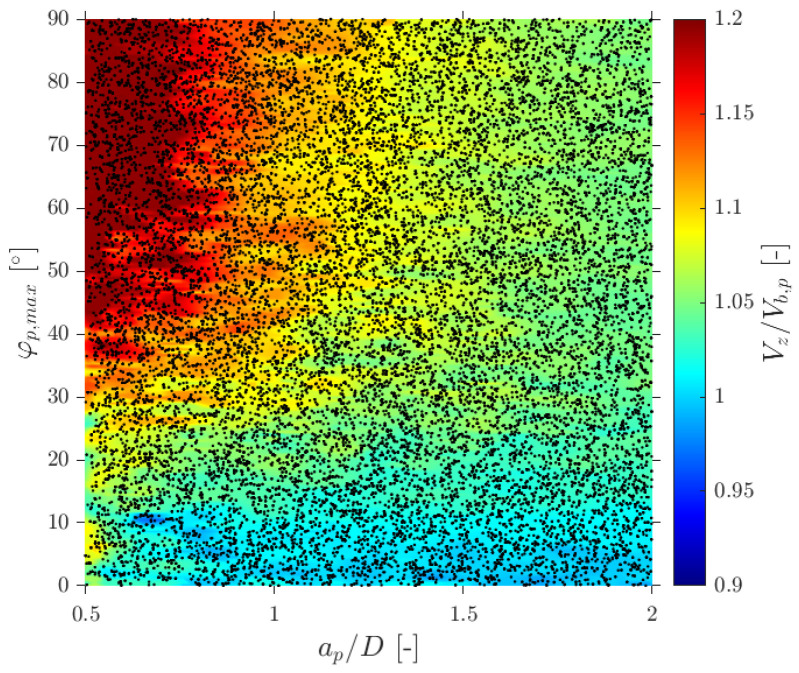
Radial bubble velocity fluctuation effect over velocity ratio.

In terms of bubble centroid velocity measurement, relative errors remain in a acceptable ± 10% interval, as depicted in Figure 30. As stated in the previous section, flux velocity is affected by bubble radial velocity fluctuation and $a_{p}/D$ ratio. Figure 31 serves to evaluate the critical values, where the $V_{z}/V_{b,p}$ ratio is mapped against $\varphi_{p,max}$.

As expected, the error is minimum if the axial flow condition is considered, $\varphi_{p,max}\leq 35^{{}^{\circ}}$. Above this value, the error is almost proportional to the $a_{p}/D$ ratio. In order to generalize results, the following correction factor based again on $m_{r}$ is proposed for flux velocity estimation:

(39) $$V_{z,corr}\left(m_{r}\right)=\frac{1}{1-0.062m_{r}+0.0062{m_{r}}^{2}}\;m_{r}\in \left[0,0.7\right]$$

**Figure 32:**
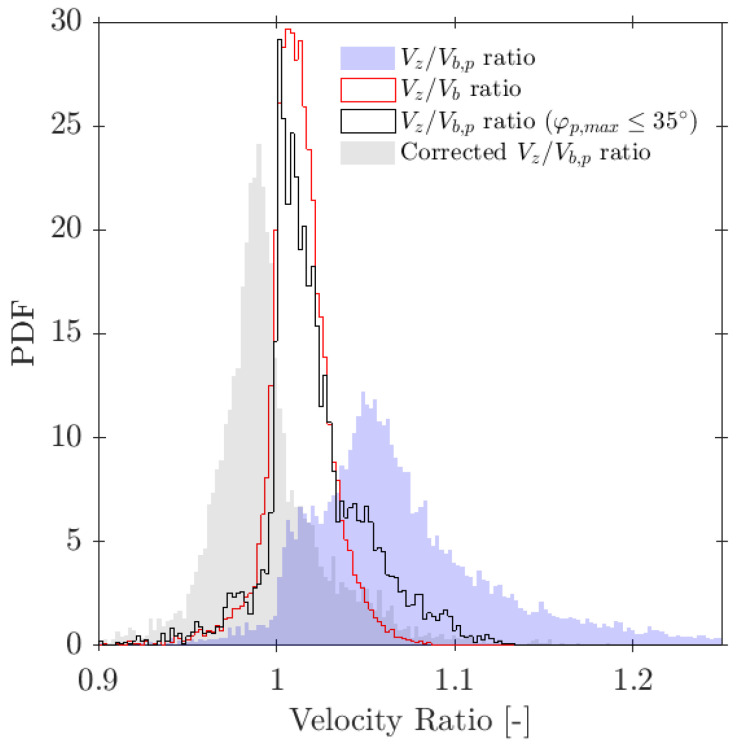
Evaluation of velocity ratios for S-Main, and proposed velocity correction for $V_{b,p}$.

Obtained velocity ratios from S-Main simulation are shown in Figure 32.

Considering the correction factor, the results of th axial flow condition and $V_{z}/V_{b,p}$ ratio are quite similar. Figure 32 also shows that the proposed correction limits and centers the relative error for $V_{b,p}$ estimation in the ± 10% range.

Region A in Table 3 corresponds to the recommended design space for velocity measurements. Within $0.5\leq a_{p}/D\leq 2$ and $r_{p}/D\leq 0.25$, the axial velocity $V_{z}$ provides the best available estimator of both the bubble centroid velocity $V_{b}$ and its projection along the probe axis $V_{b,p}$, with relative errors typically within ±10% for moderate incidence angles (Figure 31 and Figure 32). In this region, the proposed correction factor $V_{z,\mathrm{corr}}\left(m_{r}\right)$ [Equation (39)] allows the flux velocity to be recovered while keeping the error for $V_{b,p}$ centred and bounded in a similar ±10% range. Regions B–D summarize non-ideal geometries from the viewpoint of velocity measurements. Large probe radius (Region B) enhances local curvature effects and leads to strong under- or overestimation of $V_{z}$ depending on whether the bubble is pierced near its center or edge. Very small tip spacings (Region C) tend to amplify the overestimation of $V_{z}$, especially as radial velocity fluctuations increase, while very large spacings (Region D) primarily increase the missing ratio $m_{r}$ without a clear gain in velocity resolution. These non-ideal regions are therefore not recommended when accurate bubble or flux velocities are a primary objective.

### 7.4. Chord Length Measurements

As concluded in the previous sections, CL should be calculated by means of $V_{z}$ measurement, as long as $V_{z}$ is the best estimator of $V_{b}$. Four-sensor probes presumably can not provide an exact measurement of $V_{b}$ due to local bubble curvature; however, accuracy on $V_{b}$ is still reasonable although it is always overestimated to some degree, according to the proposed probe geometry limitations in Equation (38).

In general, the harmonic mean of chord length measurements tends to overestimate the corresponding bubble population chord length ($CL_{V_{z}}/CL_{th}\geq 1$), caused by positive $m_{r}$. If $m_{r}$ increases then more bubbles pierced near its edge are missed and are not taken into account in the chord length computation. However, if measured chord length is related only to computed bubbles ($CL_{V_{z}}/CL_{th}^{N_{bc}}\geq 1$), the only source of errors comes from $V_{b}$ estimation.

**Figure 33:**
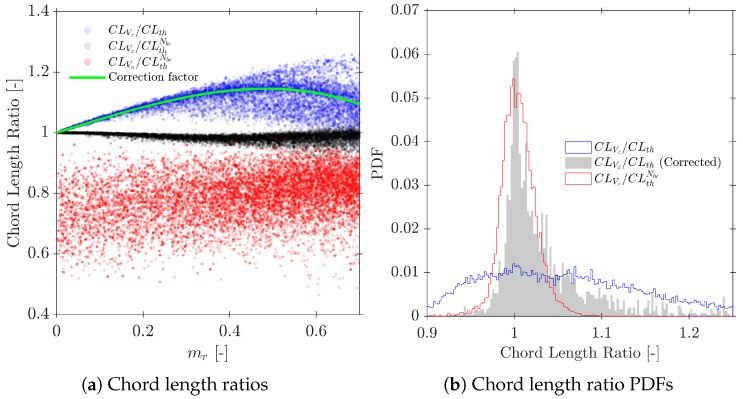
Chord length ratios and proposed correction as a function of missing ratio $m_{r}$ in the S-Main simulation. (**a**) Harmonic-mean chord length ratios for different definitions: $CL_{Vz}/CL^{th}$, $CL_{Vz}/CL_{N_{bc}}^{th}$ (computed bubbles only) and $CL_{V_{n}}/CL_{N_{bc}}^{th}$. The ratio $CL_{Vz}/CL^{th}$ increases systematically with $m_{r}$, while $CL_{Vz}/CL_{N_{bc}}^{th}$ remains within ±5%, indicating that the dominant bias comes from missed bubbles rather than from $V_{z}$ errors. The proposed correction $CL_{corr}\left(m_{r}\right)$ collapses $CL_{Vz}/CL^{th}$ back towards unity for $m_{r}\leq 0.7$. (**b**) Probability density functions of the same ratios, showing that, after correction, the chord length estimate for the full population attains a dispersion comparable to that of the computed-bubble subset.

Figure 33a depicts the mentioned chord length ratios plus chord length ratio considering $V_{n}$ ($CL_{V_{n}}/CL_{th}^{N_{bc}}$).

As stated previously, chord length can not be obtained from $V_{n}$, as it depends on interface orientation. This fact is illustrated in Figure 33b, showing the highest deviations for the $CL_{V_{z}}/CL_{th}^{N_{bc}}$ ratio. As expected, the $CL_{V_{z}}/CL_{th}^{N_{bc}}$ ratio shows deviations within a ±5% interval. Considering complete bubble population, the $CL_{V_{z}}/CL_{th}$ ratio is only unitary for $m_{r}\approx 0$, otherwise it increases with $m_{r}$.

We would suggest the following correction function, obtained by fitting the chord length ratio $CL_{V_{z}}/CL_{th}$ to a rational function, only dependent of $m_{r}$:

(40) $$CL_{corr}\left(m_{r}\right)=\frac{1.84-m_{r}}{1.84-1.84m_{r}+{m_{r}}^{2}}\;m_{r}\in \left[0,0.7\right]$$

Figure 33b shows the corresponding PDFs for the different chord length ratios. It could be stated that after correction, errors on the estimation of original bubble population are similar to those referring only to computed bubble populations.

Note that in S-Main simulation definition, $V_{b}$ (modulus) and $\varphi_{p}$ for each iteration (individual bubble) are uncorrelated. This fact, in addition to ellipsoidal bubble definition and polydisperse condition, helps to generalize chord length evaluation results. Moreover, the correction factor proposed is likely to be used in any bubbly flow condition to obtain a representative value of the chord length, without any assumption based on the bubble aspect ratio. A summary of Region A in Table 4 corresponds to the recommended design space: for $0.5\leq a_{p}/D\leq 2$ and $r_{p}/D\leq 0.25$, and as long as $m_{r}\leq 0.7$, the ratio $CL_{Vz}/CL_{N_{bc}}^{th}$ remains within a few percent of unity and the corrected chord length $CL_{corr}$ reproduces the population chord length distribution with similar accuracy. Regions B–D illustrate non-ideal geometries: a large probe radius (Region B) preferentially samples central impacts and misses short chords near the bubble edge; very small spacing (Region C) amplifies the overestimation of $V_{z}$ and thus of $CL_{Vz}$; and very large spacing (Region D) mainly increases the missing ratio without improving chord length resolution. These non-ideal regions are therefore not recommended when accurate chord length statistics are a primary objective.

For completeness, two other practical aspects that are important for experimental use of four-sensor probes are documented in the Appendices. Appendix A discusses the convergence of the Monte Carlo estimates and provides a practical guideline for the minimum number of detected bubble interfaces required to obtain reliable interfacial-area measurements in polydisperse bubbly flows. Appendix B summarizes the behaviour of the different probe layouts in near-wall conditions and outlines simple geometric recommendations for positioning the tips with respect to the wall (in particular, the advantages of the *Tetra1* configuration). The main text therefore focuses on the bulk-flow response and geometry-dependent bias, while the Appendices collect these more implementation-oriented details.

## 8. Conclusions

This work has introduced a generalized Monte Carlo framework to evaluate intrusive four-sensor probes in dispersed bubbly flows, relaxing the classical spherical-bubble and purely axial-trajectory assumptions. Bubbles are represented as spheres or ellipsoids and a wide range of non-dimensional probe geometries and incidence-angle statistics is explored. Local two-phase flow quantities (interfacial area concentration, bubble and flux velocities, chord lengths) are recovered from synthetic four-sensor signals, and the intrinsic bias associated with probe geometry and bubble–probe kinematics is quantified. The main conclusions are as follows:

- **Interfacial area concentration.** The simulations confirm that a correction for the interfacial area concentration is required whenever the missing ratio $m_{r}$ is positive, in agreement with previous works. Here, this correction is shown to remain applicable without assuming spherical bubbles and when a full distribution of radial bubble velocities is considered. Using a volumetric definition of interfacial area ensures convergence in polydisperse conditions and provides a practical criterion for the minimum number of detected interfaces in experiments. An alternative correction expressed solely in terms of $m_{r}$ is proposed, extending the applicability up to $m_{r}<1$. Within the recommended geometry range $r_{p}/D\leq 0.25$ the corrected interfacial area remains quantitatively reliable, with typical errors below 10%; for a larger probe radius (e.g., $r_{p}/D>0.3$) the underestimation of $a_{i}$ can easily exceed 20–30% due to the increase in $m_{r}$.
- **Probe geometry.** The results show that both the spacing-to-diameter ratio $a_{p}/D$ and the dimensionless probe radius $r_{p}/D$ control the measurement bias. While $a_{p}/D$ has been widely studied in the literature, the present sensitivity maps highlight the importance of $r_{p}/D$ in setting the missing ratio and curvature effects. Combining all metrics, probe geometries in the range $0.5\leq a_{p}/D\leq 2$ and $r_{p}/D\leq 0.25$ (Equation (38)) are recommended as a robust compromise between spatial resolution and accuracy for all local quantities considered. Outside this window, either very small spacing ($a_{p}/D<0.5$) or very large spacing ($a_{p}/D>2$), as well as a large probe radius ($r_{p}/D>0.25$), lead to strong increases in $m_{r}$ and to systematic biases in velocity, interfacial area and chord length estimates that readily exceed 20–30%.
- **Velocity measurements.** The analysis clarifies the role of the different velocity definitions. The interfacial velocity $V_{n}$ (Equation (13)) is mainly suitable for interfacial-area estimation. In contrast, the axial velocity $V_{z}$ (Equation (12)) emerges as the most reliable estimator of both the bubble centroid velocity $V_{b}$ and its projection along the probe axis $V_{b,p}$. Within the recommended geometric range and for moderate incidence angles (e.g., $\varphi_{p,max}\leq 30^{{}^{\circ}}$), relative errors in $V_{b}$ and $V_{b,p}$ remain typically within ±10%. When the spread of incidence angles is increased (e.g., $\varphi_{p,max}\approx 60^{{}^{\circ}}$), the velocity error can grow beyond 20% even for favourable geometries, reflecting the kinematic limitations of four-sensor probes in highly oblique bubbly flows. A compact correction factor $V_{z,corr}\left(m_{r}\right)$ is proposed to recover the flux velocity from $V_{z}$ under a wide range of bubbly flow conditions.
- **Chord length measurements.** The appropriate velocity for chord length estimation is again $V_{z}$, leading to $CL_{Vz}=V_{z}\;t_{0}$. For the subset of computed bubbles, $CL_{Vz}$ provides accurate chord lengths, consistent with what can be obtained in real experiments. When a significant fraction of bubbles is missed, $CL_{Vz}$ tends to overestimate the population-average chord length, as short chords near the bubble edge are under-sampled. A correction factor $CL_{corr}\left(m_{r}\right)$ (Equation (40)) is introduced to account for this effect using only the missing ratio. Within the recommended geometry range and for $m_{r}\leq 0.7$, this correction recovers the chord length distribution of the full bubble population with a residual bias of only a few percent, whereas for larger $m_{r}$ the chord length statistics become increasingly unreliable.
- **Near-wall configurations.** The probe arrangement with respect to the wall has a noticeable impact on the estimated local quantities. Configurations that place a rear tip too close to the wall increase the missing ratio and distort both velocity and chord length estimates. Among the tested layouts, the *Tetra1* configuration is identified as the most suitable for near-wall measurements, as it mitigates these effects and yields more balanced sampling of bubble impacts.

The modeling choices adopted in the framework (representation of bubbles as quasi-ellipsoids, locally constant centroidal velocity during each interaction, and probe rotation used as a kinematic approach for non-axial trajectories) are discussed in detail in the algorithm section. These assumptions deliberately isolate geometrical and kinematic effects, leading to a simple and computationally efficient tool to assess the intrinsic bias of four-sensor probes. Within this scope, the generalized correction curves and design maps derived in this work are intended for intrusive four-sensor phase-detection probes (conductivity, optical, capacitive, etc.) operating in dispersed bubbly flows, where bubbles can be approximated by quasi-ellipsoidal shapes. Their application is most robust for probe geometries within $0.5\leq a_{p}/D\leq 2$ and $r_{p}/D\leq 0.25$ and for missing ratios $m_{r}<0.7$. Under these conditions, the proposed correction factors enable four-sensor probes to provide quantitatively reliable estimates of interfacial area, bubble and flux velocities, and chord length distributions. Outside these ranges, the corrections should be used with caution and the probe output is better interpreted qualitatively rather than as a precise quantitative estimate of local two-phase flow parameters. The present Monte Carlo framework should therefore be understood as a theoretical performance benchmark for intrusive four-sensor probes in dispersed bubbly flows. A natural next step is to confront the generalized correction curves and design maps with dedicated experiments in well-controlled bubbly conditions, using several probe geometries within the explored $\left(a_{p}/D,r_{p}/D\right)$ ranges and combining probe signals with independent diagnostics (e.g., image-based bubble size and void fraction). Such a systematic validation lies beyond the scope of this work and is identified as an important direction for future studies.

## Figures and Tables

**Table 1:** Probe dimensions for each configuration.

	Tip	X	Y	Z
	P1	$r_{p}$	0	$a_{p}$
*Tetra1*	P2	$r_{p}\cos\left(120^{{}^{\circ}}\right)$	$r_{p}\sin\left(120^{{}^{\circ}}\right)$	$a_{p}$
	P3	$r_{p}\cos\left(240^{{}^{\circ}}\right)$	$r_{p}\sin\left(240^{{}^{\circ}}\right)$	$a_{p}$
	P1	$r_{p}$	0	$a_{p}$
*Tetra2*	P2	$r_{p}+r_{p}\cos\left(120^{{}^{\circ}}\right)$	$r_{p}+r_{p}\sin\left(120^{{}^{\circ}}\right)$	$a_{p}$
	P3	$r_{p}+r_{p}\cos\left(240^{{}^{\circ}}\right)$	$r_{p}+r_{p}\sin\left(240^{{}^{\circ}}\right)$	$a_{p}$
	P1	$2r_{p}\cos\left(45^{{}^{\circ}}\right)$	0	$a_{p}$
*Square*	P2	$2r_{p}\cos\left(45^{{}^{\circ}}\right)$	$2r_{p}\sin\left(45^{{}^{\circ}}\right)$	$a_{p}$
	P3	0	$2r_{p}\sin\left(45^{{}^{\circ}}\right)$	$a_{p}$

**Table 2:** Simulations performed.

Simulation	Cases (Iterations)	Bubble Population	$r_{p}$/*D* [-]	$a_{p}$/*D* [-]	Probe Geometry	$\varphi_{p,x}$, $\varphi_{p,y}$ [°]	Velocity *V_b_*
S-Wide	$20\times 10^{3}$ ($1\times 10^{4}$)	S/M	[0, 0.45]	[0.1, 4]	T1,S	BG/[0, 90]	CNT
S-Geom	$35\times 10^{3}$ ($3\times 10^{4}$)	S/P	[0, 0.45]	[0.1, 4]	T1,T2,S	BG/[0, 90]	CNT
S-Main	$65\times 10^{3}$ ($3\times 10^{4}$)	S,E/M,P	[0, 0.25]	[0.5, 2]	T1,T2,S	BG,U/[0, 90]	G,CNT
S-Wall	$65\times 10^{3}$ ($3\times 10^{4}$)	S,E/M,P	[0, 0.25]	[0.5, 2]	T1,T2,S	BG/[0, 90]	G

S: Spherical bubbles, E: Ellipsoidal bubbles. M: Monodisperse, P: Polydisperse. T1: Tetra1, T2: Tetra2, S: Square. BG: Bivariant Gaussian PDF, G: Gaussian PDF, U: Uniform PDF, CNT: constant (unitary modulus).

**Table 3:** Bubble velocity measurement performance as a function of non-dimensional probe geometry.

Region	Geometry Range	Velocity Behaviour
A—Recommended	$0.5\leq a_{p}/D\leq 2$; $r_{p}/D\leq 0.25$	$V_{z}$ best estimator of $V_{b}$; errors typically ±10% for moderate angles; Equation (39) applicable.
B—Large probe radius	$r_{p}/D>0.25$ (any $a_{p}/D$)	Strong curvature effects; $V_{z}$ under- or overestimated depending on impact position and probe arrangement.
C—Very small spacing	$a_{p}/D<0.5$	Systematic overestimation of $V_{z}$ (and $V_{b,p}$); more sensitive to radial velocity fluctuations.
D—Very large spacing	$a_{p}/D>2$	Higher $m_{r}$; flux velocity becomes more sensitive to incidence-angle distribution.

**Table 4:** Chord length measurement performance as a function of non-dimensional probe geometry.

Region	Geometry Range	Chord Length Behavior
A—Recommended	$0.5\leq a_{p}/D\leq 2$; $r_{p}/D\leq 0.25$	$CL_{Vz}/CL_{N_{bc}}^{th}\approx 1$ for $m_{r}\leq 0.7$; Equation (40) applicable.
B—Large probe radius	$r_{p}/D>0.25$ (any $a_{p}/D$)	Higher $m_{r}$; short chords near the bubble edge are missed; strong geometry-induced bias.
C—Very small spacing	$a_{p}/D<0.5$	Systematic overestimation of $V_{z}$ and thus of $CL_{Vz}$; increased sensitivity to incidence angles.
D—Very large spacing	$a_{p}/D>2$	Larger $m_{r}$ without gain in chord length resolution.

**Figure A1:**
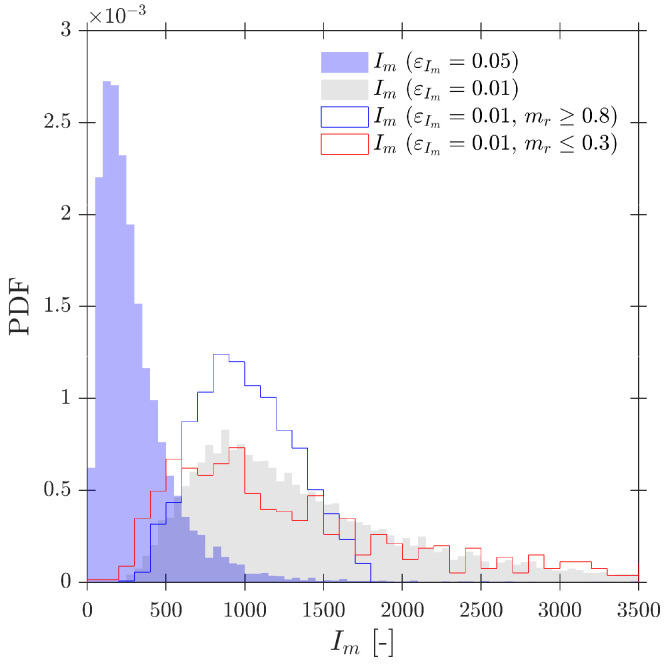
Minimum computed bubbles necessary for $a_{i,m}$ convergence.

**Figure A2:**
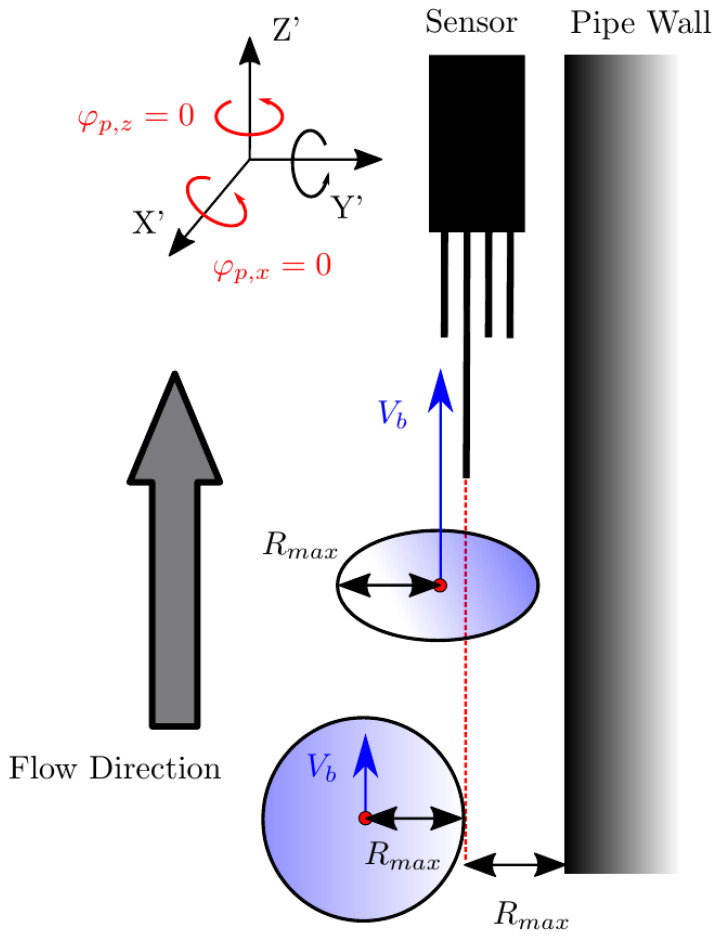
Near-wall measurements.

**Figure A3:**
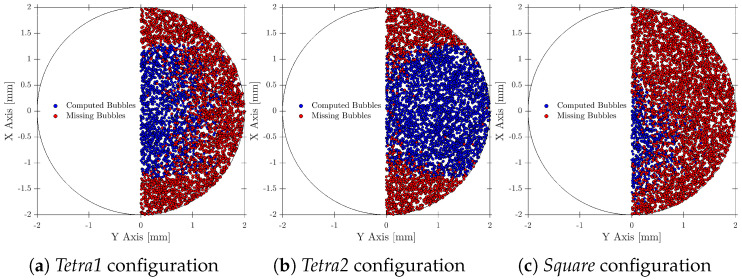
Probe geometry effect over computed/missing bubble impact point area distribution.

**Figure A4:**
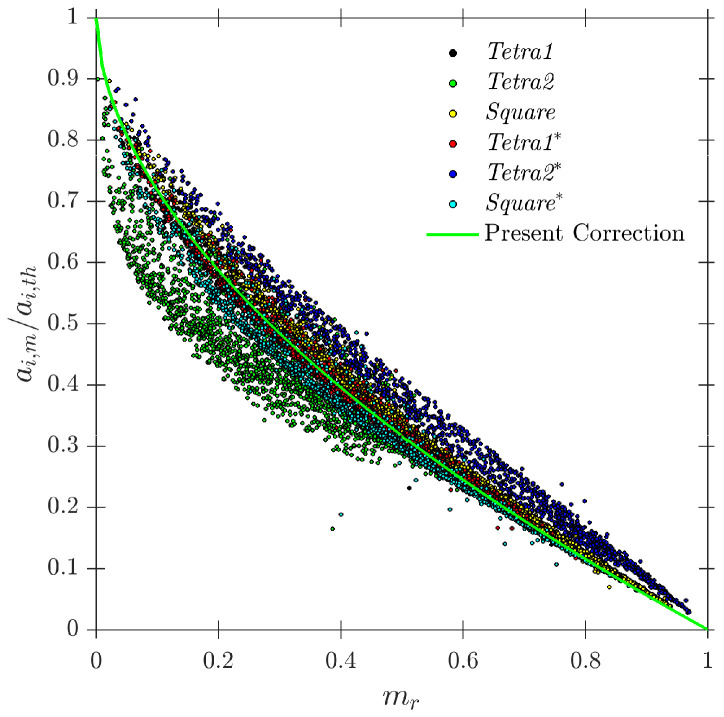
$a_{i,m}/a_{i,th}$ against $m_{r}$ for tested geometries.

**Figure A5:**
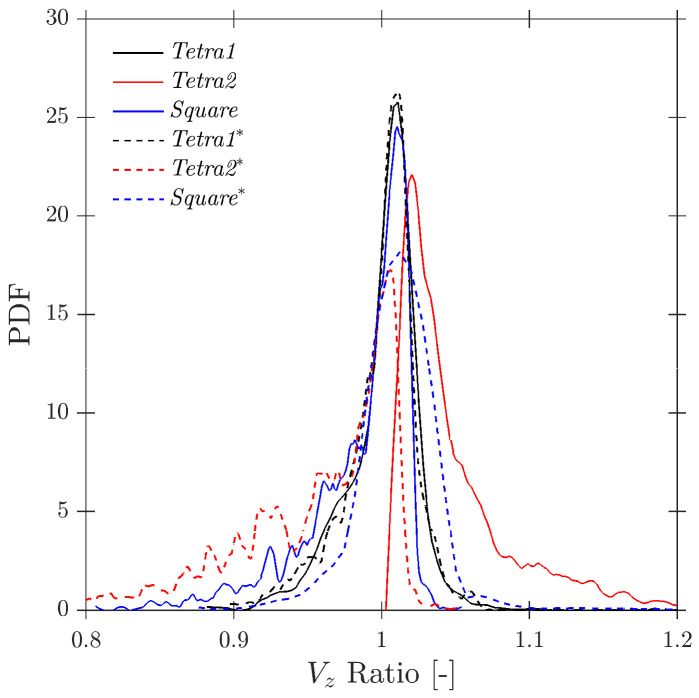
Velocity relative errors for tested geometries.

## Data Availability

The results presented in this article summarize all relevant findings of the study. The underlying datasets and complementary materials are available from the corresponding author upon request.

## Abbreviations

**Time/phase descriptors**	
*T*	Total measurement time interval (s).
τ	Total gas residence time within *T* (s).
α	Local time-averaged void fraction (-).
$\tau_{1,s}$, $\tau_{1,e}$	Start/end detection times at the front tip (sensor 1) (s).
$t_{0}$	Front-tip flight (residence) time inside a bubble, $t_{0}=\tau_{1,e}-\tau_{1,s}$ (s).
$t_{0,i}$	Front-tip flight time for the *i*-th bubble (s).
$t_{12},t_{13},t_{14}$	Time delays between front tip and rear tips 2–4 (s).
Φ	Vector of measurable delays, $\Phi =(t_{0},t_{12},t_{13},t_{14})$.
**Velocities**	
$V_{b}$	Bubble centroid velocity and magnitude (m s^−1^).
$V_{z}$	Axial (probe-axis) velocity estimate from time delays (m s^−1^).
$V_{n}$	Interfacial (transport) velocity from four-sensor probe (s^−1^).
$V_{n}^{*}$	Exact interfacial velocity using surface normal (numerical reference) (m s^−1^).
$n_{j}$, $\theta_{j}$	Local unit normal and angle with transport direction at the *j*-th contact point (°).
**Chord length (CL)**	
CL	Exact chord length in the centroid direction, $CL=V_{b}\;t_{0}$ (m).
$CL_{Vz}$	Chord length from axial estimate, $CL_{Vz}=\left|V_{z}\right|\;t_{0}$ (m).
$CL_{Vn}$	Chord length from interfacial estimate, $CL_{Vn}=\left|V_{n}\right|\;t_{0}$ (m).
$CL_{th}$	Ideal chord length for all simulated bubbles, $CL_{th}=V_{b}\;t_{0,i}$ (m).
$CL_{th}^{N_{bc}}$	Ideal chord length restricted to computed bubbles ($N_{bc}$) (m).
**Interfacial Area Concentration (IAC)**	
Γ(t)	Instantaneous global (volumetric) IAC (m^−1^).
$a_{i}\left(P,t\right)$	Local interfacial area concentration at point P(x,y,z) (m^−1^).
$a_{i,th}$	Volumetric IAC from bubble-wise ratios (m^−1^).
$a_{i,th}^{*}$	Ideal local IAC using $V_{n}^{*}$ (local-definition reference) (m^−1^).
$a_{i,m}$	Measured local IAC from four-sensor probe using $V_{n}$ (computed bubbles only) (m^−1^).
$a_{i,mc}$	Curvature-corrected local IAC using $V_{n}^{*}$ (computed bubbles only) (m^−1^).

**Probe orientation / angular parameters**	
$\varphi_{p}$	Probe rotation-angle vector, $\varphi_{p}=\left[\varphi_{p,x}\;\varphi_{p,y}\;\varphi_{p,z}\right]$ (rad or °).
$\varphi_{p,x}$, $\varphi_{p,y}$	Probe inclination angles (set the probe attacking direction) (rad or °).
$\varphi_{p,z}$	Probe rotation about its own $Z^{\prime }$ axis (rad or °).
$\varphi_{p,max}$	Maximum bubble attacking angle used to build the distribution of $\varphi_{p}$ (rad or °).
**Counting / missing events**	
$N_{b}$	Total number of simulated bubbles/events (-).
$N_{bc}$	Number of computed (detected) bubbles by the four-sensor probe (-).
$m_{r}$	Missing bubble ratio, $mr=1-N_{bc}/N_{b}$ (-).
**Probe and geometry**	
a,b,c	Semi-axes of an ellipsoidal bubble (m).
*D*	Equivalent diameter, $D=2\sqrt[{3}]{abc}$ (m).
ap, rp	Axial and radial spacing between rear tips and the front tip (m).
ap/D	Axial probe spacing normalized by the equivalent bubble diameter (–).
rp/D	Radial probe spacing normalized by the equivalent bubble diameter (–).

## Appendix A. Theoretical Minimum Number of Sampled Bubbles for Accurate Measurements

As previously stated in Section 4.4, the evaluation of the convergence for $a_{i,m}$ measurements would also provide the minimum interfaces, $I_{m}$, to be detected in order to theoretically provide a stable value for measured interfacial area. S-Main simulation provides a large amount of different flow simulations to obtain insight about the needed computed bubbles in real experiments.

Figure A1 depicts the $I_{m}$ PDFs considering different admissible relative errors, εIm, and $m_{r}$. As long as $a_{i,m}$ only depends on computed bubble information, $m_{r}$ influence is negligible. However, the admissible εIm is important for determining $I_{m}$. According to data shown in Figure A1, in general, at least 1000 interfaces, $I_{m}$, should be detected to theoretically obtain a proper value for measured interfacial area, considering an admissible εIm≤5%. This $I_{m}$ could be used to limit the acquisition time in real experiments.

As long as $a_{i,m}$ only takes into account computed bubbles, the PDF shape obtained in Figure A1 is almost invariant regardless of changes in probe orientation (not dependent on $\varphi_{p,max}$) and bubble velocity definition (not dependent on $V_{b}$ modulus).

## Appendix B. Near-Wall Measurements

In a vertical pipe two-phase flow, specifically near-pipe wall measurements require specific considerations. Velocity gradients are higher and bubble detection could not be homogeneous in this zone. As depicted in Figure A2, if a sensor is located a distance less than the mean bubble radius, there are two main problems regarding how the bubble surface gradients affect the measurements. On one hand, the mean bubble radius will always be underestimated [42] if $r_{p}/D<Rmax$. On the other hand, velocity measurements will be affected due to limited probe-interface possible orientation (bubbles tend to be pierced on the same bubble side).

In terms of simulation definition, the presence of the wall limits the possible approaching direction of bubbles; therefore, probe axis rotation (defined by $\varphi_{p,max}$ and $\varphi_{p,z}$) can not be considered as a random variable as in other pipe radial positions. This fact suggests that probe geometry (rear tip arrangement) would have an important influence over measurable flow parameters as the relative probe-to-wall orientation should be kept constant.

For the numerical simulations performed for this specific situation, S-Wall (see Table 2), we will consider the following assumptions and considerations:

- We will consider an arbitrary bubble size generation (spherical/ellipsoidal bubbles). However, in order to limit the front sensor tip position between wall and a bubble radius ($R_{max}$) distance from the wall, *a* and *b* has are considered equal and constant for all generated bubbles. Thus, the only bubble size source of variability is included in *c*.
- We consider $\varphi_{p,x}=\varphi_{p,z}=0$. These conditions ensure that the probe maintains its orientation for all bubble–probe interactions and avoid unrealistic effects such as a bubble moving throughout the wall. $\varphi_{p,y}$ has been left intentionally as a source of variability as long as the lateral motion of the bubbles in the X-axis does not interfere with the wall.
- We have used the probe geometrical arrangement defined in Table 1 and three extra probe orientations redefined by means of a probe Z’-axis rotation in order to provide more information about the influence of probe–wall orientation.

Figure A3 illustrates the performance of the three probe geometries defined in Table 1 for a specific case with $\varphi_{p,y}=20^{{}^{\circ}}$, $a_{p}/D=1$, and $r_{p}/D=0.1$. As expected, it illustrates a different response among the probe configurations.

For *Tetra1* geometry, computed bubbles are mostly caught in the center, which in most cases corresponds to minimum surface curvature. This suggests that errors caused by curvature will be minimized. For *Tetra2* geometry, computed bubbles cover almost all surfaces. As the probe orientation is constant (rear sensors oriented towards the center of the bubble) important errors due to bubble curvature are expected, as the condition $t^{*}>t_{01},t_{02},t_{03}$ will be the common one. Performance for *Square* geometry appears to be very similar to *Tetra1* geometry, where computed bubbles tend to be caught near the bubble center. However, the $m_{r}$ appears to be higher.

For sake of generality, S-Wall considers three extra probe sensor orientations by means of a simple rotation of the Z’-axis: *Tetra1** obtained from a 90° clockwise rotation of *Tetra1*, *Tetra2** obtained from a 180° rotation of *Tetra2*, and *Square** obtained from a 45° clockwise rotation of *Square*.

Figure A4 shows the results from S-Wall simulation in terms of the $a_{i,m}/a_{i,th}$ ratio.

Several differences can be observed with respect to the isotropic simulations (S-Main and S-Geom). Despite different probe configurations, the $a_{i,m}/a_{i,th}$ ratio is in agreement with the proposed correction for interfacial area measurement, except for *Tetra2* and *Tetra2** geometries. For these two geometries, up to 20% relative errors can be found from the proposed correction factor. Figure A4 also depicts the better accuracy of the correction factor for high $m_{r}$. This is explained by the fact that, for near-wall conditions, and specially for *Tetra2* and *Tetra2**, computed bubbles will tend to be mainly pierced near their maximum axial radius, which is the same situation as if we consider the isotropic bubble–sensor interactions depicted in the previous Sections.

Similar conclusions as for the measured interfacial area could be obtained for measured velocity $V_{z}$ by means of the $V_{z}/V_{b,p}$ ratio, as depicted in Figure A5.

A similar Vz ratio PDF is obtained for *Tetra1* and *Tetra1**, providing the best $V_{b,p}$ estimation with the lowest deviation among all other probe configurations and orientations. The two possible explanations are as follows: (1) as depicted in Figure A3a, missing bubbles are mostly caught near its edge, therefore $V_{z}$ is accurately computed only in low curvature bubble surface points; (2), as these configurations are based on a symmetric distribution of the rear sensors with respect to the front one, they are less influenced by changes in the probe orientation. For the other configurations, as more rear sensors are located further from the front sensor, local bubble curvature has increasing influence and probe orientation also becomes important. For *Square* and *Square** geometries, measured $V_{z}$ tends to underestimate $V_{b,p}$. *Tetra2* and *Tetra2** present the highest deviations for $V_{b,p}$ estimation. In these cases, the probe orientation is critical for $V_{z}$ measurement as it can cause the overestimation or underestimation of $V_{b,p}$.

The conclusion from these observations is that, for near-wall velocity measurements, probe geometry and the rear probe tips with respect to the wall are very important. The same considerations can be stated for chord length, as its accurate measurement also relies on $V_{b,p}$. It is recommended to use as symmetrical as possible a distribution of the rear sensors around the front sensor as in *Tetra1* to obtain the best accuracy. Considering the interfacial area recovery, it is important to remark that using the correction factor proposed by [14] or the similar one proposed in this work is still accurate.
